# Effects of digital health interventions on muscle mass, muscle strength, and physical function in older adults with sarcopenia: a systematic review and meta-analysis

**DOI:** 10.3389/fpubh.2025.1711514

**Published:** 2025-11-26

**Authors:** Wenjia Chen, Jiayi Yao, Haozhe Wang, Shiguan Jia, Xueqiang Zhu, Lihong Mao

**Affiliations:** 1 School of Physical Education, China University of Mining and Technology, Xuzhou, China; 2 School of Competitive Sport, Shandong Sport University, Rizhao, China

**Keywords:** digitalization, sarcopenia, older adults, muscle mass, muscle strength, physical function, meta-analysis, randomized controlled trials (RCTs)

## Abstract

**Background and aims:**

Sarcopenia, an age-related progressive muscle disorder, is characterized by low muscle strength. While digital health technologies are emerging as a management tool, systematic evidence of their comprehensive effects on older adults diagnosed with sarcopenia is lacking. We therefore aimed to comprehensively evaluate the effects of digital health interventions (DHIs) on muscle mass, muscle strength, physical function, and quality of life in this specific population.

**Methods:**

We systematically searched PubMed, Embase, Web of Science, and the Cochrane Library for randomized controlled trials (RCTs) published up to 13 September 2025. Eligible patients were aged ≥60 years with a formal diagnosis of sarcopenia. We conducted a meta-analysis to assess intervention effects and used the GRADE system to assess the certainty of evidence.

**Results:**

Eleven RCTs with a total of 757 patients were included. The meta-analysis revealed that DHIs significantly improved skeletal muscle mass [Standardized Mean Difference (SMD) = 0.35, 95% CI: 0.13–0.57] and grip strength (SMD = 0.28, 95% CI: 0.04–0.53). However, improvements in physical function were selective, while indicators such as sit-to-stand time improved, no significant effects were found for gait speed, walking distance, or activities of daily living (ADL). The effect on quality of life (QoL) was uncertain. The certainty of evidence was moderate for skeletal muscle mass and low for grip strength.

**Conclusion:**

Digital health interventions appear effective in improving muscle mass and muscle strength in older adults with sarcopenia, though their impact on physical function is selective. Technologies that provide real-time interaction and personalized feedback, particularly those based on artificial intelligence (AI) and virtual/mixed reality (VR/MR), are promising, although current evidence is preliminary. From a public health standpoint, the scalability and accessibility of DHIs represent a valuable supplementary strategy for sarcopenia management.

**Systematic review registration:**

www.crd.york.ac.uk/prospero, identifier CRD420251151435.

## 1 Introduction

Sarcopenia is an age-related progressive muscle disease, with low muscle strength as the core criterion for its diagnosis, confirmed by low muscle mass or low physical function according to international consensus (e.g., EWGSOP2, AWGS) (1, 2). Sarcopenia is closely associated with a range of adverse health outcomes, including increased fall risk, disability, prolonged hospitalization, and higher mortality (3, 4). Falls themselves are a growing public health concern globally, particularly prevalent among older adults in long-term care facilities (5). In the older adult population, the negative impact of sarcopenia on physical function is multifaceted, and other age-related issues (such as poor posture) can exacerbate functional decline. For instance, a study on older adults demonstrated that a “sway-back” posture accompanied by chronic low back pain is associated with functional limitations, not only increasing fall risk but also significantly worsening gait parameters such as speed and stride length (6). Therefore, the core muscle strength decline in sarcopenia, combined with these composite factors, places this specific population at an extremely high risk of functional limitation and falls, highlighting the urgent need for effective interventions for this fundamental problem.

Exercise combined with nutritional supplementation is the current evidence-based strategy for sarcopenia management (7, 8). However, traditional face-to-face intervention models are often challenged in practice by factors such as uneven distribution of medical resources, transportation difficulties, patient mobility limitations, and financial burdens, leading to poor long-term adherence (9). Research indicates that among individuals over 75, only 9% of men and 4% of women meet the guidelines for muscle-strengthening activities (10). The COVID-19 pandemic further underscored the urgent need to develop remote health services (11). Against this backdrop, Digital Health Interventions (DHIs) have emerged. In this study, DHIs are defined as interventions that utilize information and communication technologies—such as mobile applications, wearable devices, telehealth platforms, virtual reality (VR), or exergames to support or directly provide health-related services (12, 13). They offer innovative solutions to overcome the barriers of traditional interventions. Previous evidence has shown that DHIs have the potential to improve muscle strength, functional capacity, and quality of life in the general older population (14, 15), and that remote home-based exercise conducted via video conferencing can be comparable to traditional face-to-face training in improving body composition and lower limb strength (16).

However, despite the increasing application of DHIs in sarcopenia management, significant research gaps persist. As noted in existing systematic reviews, current digital interventions (like mHealth) primarily focus on improving physical activity (PA) and body composition (BMI), while interventions targeting muscle function and strength remain a notable gap (17, 18). Previous studies often included mixed populations (e.g., healthy older adults or pre-frail individuals), lacking a comprehensive assessment specifically for the high-risk group of diagnosed sarcopenia. Furthermore, many studies focused only on single outcomes (e.g., muscle strength) and failed to systematically evaluate the overall impact of DHIs on the core components of sarcopenia (muscle mass, muscle strength, multidimensional physical function) and quality of life. Finally, the application of emerging technologies such as artificial intelligence (AI) and virtual/mixed reality (VR/MR) in sarcopenia intervention is increasing, but their effectiveness lacks systematic, high-quality evidence.

Therefore, this systematic review and meta-analysis aim to comprehensively evaluate the combined effects of various digital health interventions on muscle mass, muscle strength, physical function, and quality of life in older patients with diagnosed sarcopenia. This study seeks to fill the aforementioned research gaps and provide more precise evidence-based support for clinical practice.

## 2 Materials and methods

This study’s protocol was registered with PROSPERO (Registration No.: CRD420251151435), specifying the main objectives, inclusion and exclusion criteria, interventions, control measures, and the primary and secondary outcomes planned for assessment. The implementation of this systematic review strictly adhered to the pre-registered protocol without major deviations. It was conducted and reported in strict accordance with the Preferred Reporting Items for Systematic Reviews and Meta-Analyses (PRISMA 2020) checklist (19).

### 2.1 Search strategy

This study systematically searched PubMed, Embase, CINAHL, Web of Science, Scopus, Cochrane Library, ClinicalTrials.gov, China National Knowledge Infrastructure (CNKI), Wanfang Database, and VIP Database from their inception to 13 September 2025. The search employed a strategy combining subject headings (e.g., MeSH Terms) and free-text words, structured around four core concepts: (1) Sarcopenia (e.g., “sarcopenia,” “muscle wasting,” “muscle atrophy”); (2) Older adults (e.g., “Aged,” “older adult”); (3) Digital health interventions (e.g., “telemedicine,” “mobile app,” “Virtual Reality,” “Wearable”); and (4) Randomized controlled trials (e.g., “randomized controlled trial”). Search strategies were adapted according to the rules of each database. The complete search strings for all databases have been moved to the appendix (see Supplementary material 1). Additionally, we manually searched the reference lists of included articles to identify any omissions.

### 2.2 Inclusion and exclusion criteria

Two reviewers independently screened the titles and abstracts of the literature, followed by a full-text review of potentially eligible articles to determine final inclusion. Any disagreements were resolved through discussion or third-party arbitration. Inclusion criteria were set based on the PICO-S framework: (1) Population (P): Patients aged ≥60 years and diagnosed with sarcopenia according to internationally recognized criteria (e.g., AWGS, EWGSOP2). (2) Intervention (I): Any form of digital health technology intervention. (3) Comparison (C): Usual care, health education, traditional face-to-face intervention, or no intervention. (4) Outcomes (O): At least one of the following reported: muscle mass, muscle strength, physical function, quality of life, or activities of daily living. (5) Study design (S): Randomized controlled trial (RCT). Exclusion criteria included: duplicate publications; non-original research such as conference abstracts or editorials; studies where digital technology was used only for assessment or monitoring and not as a core intervention; studies where participants had other neuromuscular diseases that could seriously affect outcome judgment; and studies with missing or unextractable key outcome data. Furthermore, articles for which the full text was unavailable were excluded, as a comprehensive quality assessment and data extraction could not be performed. This study’s search strategy focused primarily on published peer-reviewed literature and major clinical trial registries. We did not systematically search gray literature or preprint servers. The literature search was mainly limited to English and Chinese articles. To assess potential language bias, we also searched databases in other major languages (e.g., German, French, and Japanese) but found no additional RCTs that met the inclusion criteria. Therefore, the final analysis was limited to Chinese and English literature.

### 2.3 Data collection

Two researchers independently conducted literature screening and data extraction, starting with an initial screening of titles and abstracts, followed by obtaining full texts for a second screening to finalize inclusion. Any discrepancies were resolved by discussion or consultation with a third party. A pre-designed data extraction form was used to extract the following information: (1) Basic information: first author, year of publication; (2) Population characteristics: sample size, age, sarcopenia diagnostic criteria; (3) Intervention characteristics: type of digital health technology, specific intervention content (including exercise type, prescription, frequency, duration, progression principles), supervision and feedback methods, and control group measures; (4) Outcome indicators: mean and standard deviation of skeletal muscle mass, skeletal muscle index, grip strength, walk tests, sit-to-stand tests, timed up-and-go tests, quality of life scores, and activities of daily living scores at various measurement points. For studies with incomplete data, attempts were made to contact the original authors; if unavailable, data were converted or estimated using methods recommended by the Cochrane Handbook.

### 2.4 Risk of bias and certainty of evidence

The Cochrane Collaboration’s recommended Risk of Bias tool 2.0 (RoB 2) was used to assess the quality of included RCTs (20). The assessment covered five domains: bias arising from the randomization process, bias due to deviations from intended interventions, bias due to missing outcome data, bias in measurement of the outcome, and bias in selection of the reported result. Each domain was judged as “low risk,” “some concerns,” or “high risk,” leading to an overall risk of bias judgment for each study. The GRADE system was used to evaluate the certainty of evidence (21, 22), assessing downgrades across five areas: risk of bias, inconsistency, indirectness, imprecision, and publication bias. The evidence quality was classified into four levels: High, Moderate, Low, or Very Low. Two reviewers independently completed the quality assessment; disagreements were resolved through discussion, with third-party expert consultation if necessary.

### 2.5 Data analysis

RevMan 5.4 software was used for the meta-analysis. Continuous variables were analyzed using the Standardized Mean Difference (SMD) and its 95% Confidence Interval (CI) as the effect size. For physical function indicators measured in time units (e.g., TUGT, sit-to-stand time), a negative SMD value indicates improvement (time reduction). For studies reporting data at multiple time points or multiple subgroups, the data were merged using the following formulas: Combined Mean = (*n*₁ × mean₁ + *n*₂ × mean₂)/(*n*₁ + *n*₂); Combined SD = √[((*n*₁-1) × SD₁^2^ + (*n*₂-1) × SD₂^2^)/(*n*₁ + *n*₂-2)]. The *χ*^2^ test and *I*^2^ statistic were used to assess heterogeneity. If *p* > 0.10 and *I*^2^ ≤ 50%, heterogeneity was considered acceptable, and a fixed-effect model was used. If *p* ≤ 0.10 or *I*^2^ > 50%, significant heterogeneity was present, and a random-effects model was used (23). To explore potential sources of heterogeneity for results with *I*^2^ > 50%, we pre-designed subgroup analyses based on intervention duration and technology interactivity (24, 25). Sensitivity analysis, by sequentially removing individual studies, was used to identify sources of heterogeneity. After removing the source of heterogeneity, the *I*^2^ value for most indicators dropped to 0% or within an acceptable range, indicating the results were robust. In this review, the number of studies included in the meta-analysis for each outcome was less than 10, and therefore, funnel plots were not used to assess publication bias (26).

## 3 Results

### 3.1 Study selection

**Figure 1:**
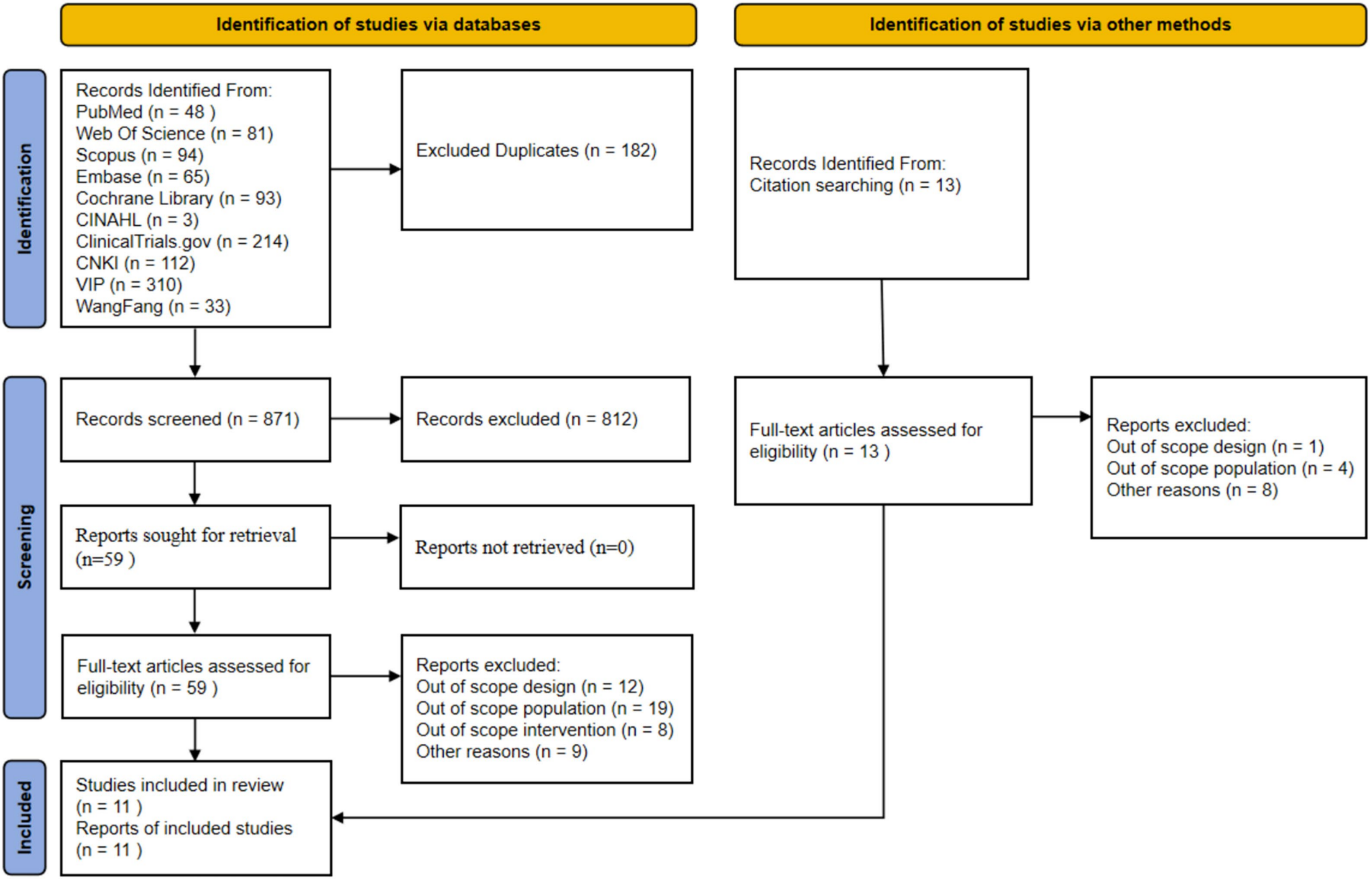
Preferred reporting items for systematic reviews and meta-analysis (PRISMA) study flow diagram. This figure illustrates the complete screening process from database retrieval to the final inclusion of 11 RCTs. Detailed screening criteria are described in the Methods section 2.2, and a detailed narrative of the screening results is provided in the Results section 3.1.

The initial search yielded 1,053 articles, including 48 from PubMed, 81 from Web of Science, 94 from Scopus, 65 from Embase, 93 from Cochrane Library, 3 from CINAHL, 214 from ClinicalTrials.gov, 112 from CNKI, 310 from Wanfang, and 33 from VIP. An additional 13 articles were supplemented through citation tracking. After removing 182 duplicates using EndNote software, 871 articles remained for title and abstract screening. A total of 812 articles that clearly did not meet the inclusion criteria were excluded. The full texts of the remaining 59 articles were obtained for detailed assessment. In the full-text assessment stage, 48 articles were excluded for the following reasons: 12 for non-compliant study design (non-RCT), 19 for non-compliant study population (not sarcopenia patients or age criteria not met), 8 for non-compliant intervention measures (not digital health intervention or used only for assessment), and 9 for other reasons (incomplete data, unextractable) (see Supplementary Table 1). Ultimately, 11 randomized controlled trials were included for systematic review and meta-analysis. The literature screening flow diagram is shown in Figure 1.

### 3.2 Study characteristics

The 11 included RCTs were published between 2017 and 2025 (27–37), as shown in Table 1. Among them, five were published in 2025 (30, 31, 34, 36, 37), three in 2024 (27, 33, 35), and one each in 2023 (28), 2022 (29), and 2017 (32). The total sample size was 757, with individual study sample sizes ranging from 23 to 142 (28, 32). All participants were older adults aged 60 and over diagnosed with sarcopenia, with mean ages ranging from 69 to 82 years (31, 32). Regarding diagnostic criteria, most included studies used internationally recognized standards. Specifically, most studies (nine studies) used the revised 2019 criteria from the Asian Working Group for Sarcopenia (AWGS), one study used the second version of the European Working Group on Sarcopenia in Older People (EWGSOP2) criteria (29), and another, although not explicitly citing a specific standard (32), based its diagnosis on reduced muscle mass and function, ensuring homogeneity among the study populations. In terms of study design, 8 were two-group parallel-controlled trials, and 3 were three-group parallel-controlled trials (27, 30, 37). The control groups typically received usual care, health education, or traditional face-to-face interventions.

**Table 1:** Characteristics of the included studies.

Author, year	Sample size (E/C)	Age (years, E/C)	Intervention (E/C)	Technology/equipment	Exercise type	Exercise prescription (intensity/volume)	Progression principle	Supervision and feedback	Outcome measures
Wang et al. (29)	114 (60/54)	70.16 ± 4.32/69.88 ± 3.29	Remote nutrition and exercise guidance based on a mobile application (APP)/conventional health education	Self-developed health management APP	Comprehensive (aerobic, resistance)	Two sessions/week, 70–90 min/session, moderate-to-high intensity, 12 weeks	N/A	Automatic feedback and reminders from the APP, no manual intervention	BMM, ASMI, grip strength, 4-m timed walking test, timing sitting and standing test
He et al. (27)	70 (24/23/23)	73.67 ± 4.77/72.26 ± 4.43/70.91 ± 3.94	AI-based remote training group/Conventional remote training/Face-to-face traditional intervention	Local computer, Tencent Meeting software	Aerobic exercise (24-form Tai Chi)	Three sessions/week, 40 min/session, low intensity, 12 weeks	Phased learning (weeks 1–4 for basics, weeks 5–12 for reinforcement)	AI real-time text-based error correction combined with follow-along video	ASMI, grip strength, 6-m walking pace, TUGT, QOL
Yin et al. (28)	142 (72/72)	72.14 ± 5.06/ 72.49 ± 5.24	Internet-based health management platform/ Conventional care	An internet and hospital management platform developed by the hospital’s information department	Comprehensive (aerobic, resistance, balance)	Two sessions/week, 12 weeks	N/A	Combined offline (1/month) and online (weekly) guidance	ASMI, grip strength, ADL
Hong et al. (32)	23 (11/12)	82.2 ± 5.6/81.5 ± 4.4	Video conference-based remote supervised resistance training/usual activities	15-inch all-in-one PC, Skype video conferencing software, 10 Mbps broadband network	Resistance training (8 exercises)	Three sessions/week, 20–40 min/session, 12 weeks, intensity: RPE 13–16	Progressive load and duration (weeks 1–4 no load; weeks 5–8 1 kg; weeks 9–12 2 kg)	Real-time one-on-one video guidance and correction	TSM, 30SSRT, TUGT
An et al. (30)	30 (15/15)	76.24 ± 8.7/74.88 ± 9.1	Mixed reality physiotherapy/conventional physical activity	Meta Quest head-mounted MR device, proprietary Mr. PT software platform	Cognitive-motor dual-task training	Three sessions/week, 30 min/session, 4 weeks	Automatic difficulty adjustment (system adjusts based on success rate)	AI real-time verbal and visual feedback combined with on-site therapist supervision	SF-12, KIADL
Chitjamnogchai et al. (31)	53 (26/27)	69.23 ± 4.79/71.15 ± 5.94	Home-based virtual reality (VR) combined training/conventional health education	Mini Android box, computer, optical heart rate sensor, software APP	Comprehensive (aerobic, resistance)	Three sessions/week, 60 min/session, 12 weeks, Aerobic intensity: 40%–59% HRR, Resistance intensity: 60%–70% 1RM	N/A	Real-time heart rate monitoring combined with follow-along video	6MWT
Ho et al. (33)	58 (27/31)	70.26 ± 4.72/74.26 ± 6.30	Wearable activity tracker (WAT)-based progressive goal-setting walking intervention/conventional health education	Asus VivoWatch BP wearable device, companion smartphone APP, Garmin Connect/Apple HealthKit data synchronization platform	Aerobic exercise (walking)	Daily activity, 8 weeks	Phased goal progression (weeks 1–4: 5,000 steps/day; weeks 5–8: 7,500 steps/day)	Device feedback with APP recording combined with weekly offline check-ups	SMI, grip strength, Timing sitting and standing test
Wu et al. (34)	80 (40/40)	≥65	Wearable device-based structured walking intervention/usual activities	Garmin Vivosmart HR, Apple Watch, Garmin Connect/Apple HealthKit data synchronization platform	Aerobic exercise (structured walking)	Five sessions/week, 30 min/session, 12 weeks, intensity: 100 steps/min (moderate intensity)	N/A	Real-time device feedback (reminder when pace is below target) with automatic data synchronization	SMM, ASMI, grip strength, 10-meter walk time, timing sitting and standing test
Tuan et al. (35)	60 (30/30)	78.83 ± 7.71/78.73 ± 6.82	Exergame-based multi-component training/conventional care	Nintendo Switch console, Ring-Con, Joy-Con wireless controllers	Multi-component training (mainly resistance, aerobic, and balance exercises for upper limbs and trunk)	Two sessions/week, 50 min/session (including warm-up and cool-down), 12 weeks, Intensity: RPE 13 (somewhat hard)	Automatic difficulty adjustment: RFA system automatically adjusts progression based on player performance	Gamified feedback including in-game visual, audio, and vibration feedback	ASMM, ASMMI, grip strength, usual gait speed
Zhang et al. (36)	51 (24/27)	70.47 ± 6.05/69.81 ± 5.76	Mobile application-based remote resistance training/face-to-face traditional intervention	Self-developed mobile application	Resistance training (six exercises for major muscle groups)	Three sessions/week, 60 min/session (including warm-up and cool-down), 4 weeks, Intensity: RPE 12–14, Volume: 3 sets × 10 repetitions	N/A	Video guidance including in-app instructional videos and text descriptions; data upload including manual upload of RPE values post-training	TSM, SMI, grip strength, 6MWT, 30SSRT, TUGT, IADL
Wei et al. (37)	76 (27/25/24)	71.57 ± 7.24/72.56 ± 7.76/70.77 ± 8.27	Deep learning-based 3D human pose estimation technology/conventional remote training/face-to-face traditional intervention	Home computer, Tencent Meeting, BlazePose 3D pose estimation model	Aerobic exercise (Yi Jin Jing)	Three sessions/week, 40 min/session, low intensity, 12 weeks	Phased learning: (weeks 1–4 for basics, weeks 5–12 for reinforcement)	AI real-time feedback for movement correction combined with follow-along video	ASMI, grip strength, 6-m walking pace, TUGT, SF-36

BMM, body muscle mass (kg); SMM, skeletal muscle mass (kg); TSM, total skeletal muscle (kg); ASMM, appendicular skeletal muscle mass (kg); SMI, skeletal muscle index (kg/m^2^); ASMI, appendicular skeletal muscle Mass index (kg/m^2^); asmmi, appendicular skeletal muscle mass index (kg/m^2^); TUGT, timed up and go test (s); 30SSRT, 30-second sit-to-stand repeated test (s); 6-meter walking (m/s); 6MWT, 6-min walk test (m); QOL, quality of life; ADL, activities of daily living; IADL, instrumental activities of daily living; KIADL, Korean instrumental activities of daily living; SF-12, 12-item short form health survey; SF-36, 36-item short form health survey.

The digital health interventions employed in the experimental groups showed significant diversity, with varying protocols. Some studies used smartphone apps as the intervention medium; for example, Wang et al. (29) provided a 12-week comprehensive training program of moderate-to-high intensity (2 sessions/week, 70–90 min/session) via an app that relied on automated feedback and reminders. In contrast, Zhang et al. (36) focused on a 4-week resistance training program (three sessions/week) guided by in-app videos. Other studies utilized wearable devices to promote and monitor walking activities, such as Ho et al. (32), who implemented an 8-week walking intervention with progressively increasing step goals to ensure progression, and Wu et al. (34) who conducted a 12-week structured walking program at a moderate intensity of 100 steps/min, with real-time device reminders.

Some studies employed technologies offering greater real-time interactivity or artificial intelligence (AI)-driven features. Hong et al. (30) utilized Skype video conferencing to deliver real-time, one-on-one remote-supervised resistance training (three sessions/week for 12 weeks), implementing progression through gradual load increases (32). He et al. (27) and Wei et al. (37) integrated AI technology into 12-week low-intensity aerobic exercises (Tai Chi and Yi Jin Jing, respectively), using real-time text prompts or 3D human pose estimation for movement correction, and established a phased learning process. Additionally, An et al. (30) used mixed reality (MR) technology for a 4-week cognitive-motor dual-task training, with a system that automatically adjusted difficulty based on participant performance. Exergames and health management platforms were also utilized. Tuan et al. (35) used the Nintendo Switch exergame system for a 12-week multi-component training program, where the game system automatically adjusted progression and provided gamified feedback. Chitjamnogchai et al. (31) implemented a 12-week comprehensive training program using home-based virtual reality (VR) combined with real-time heart rate monitoring. Yin et al. (28) relied on a hospital-developed internet platform, combining online and offline guidance for intervention. All studies measured multiple sarcopenia-related outcomes, including muscle mass (e.g., SMM, SMI, ASMI), muscle strength (grip strength), and physical function (e.g., walking speed, sit-to-stand tests, TUGT). The overall adherence to the interventions was good, with completion rates ranging from 81.72 to 100%, and five studies achieving a 100% completion rate.

### 3.3 Risk of bias

**Figure 2:**
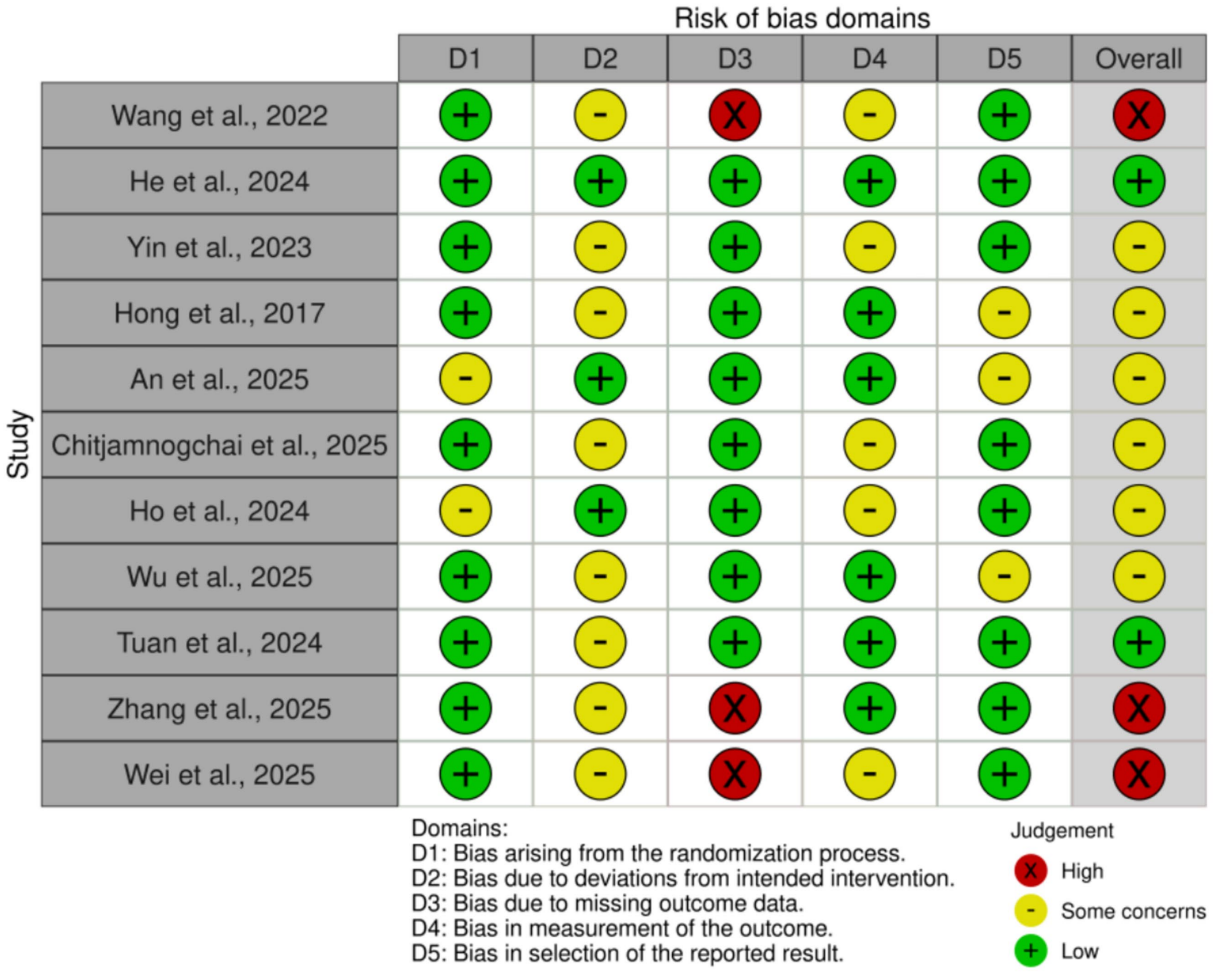
Risk of bias summary: review of the authors’ judgments about each risk of bias item for each included study.

**Figure 3:**
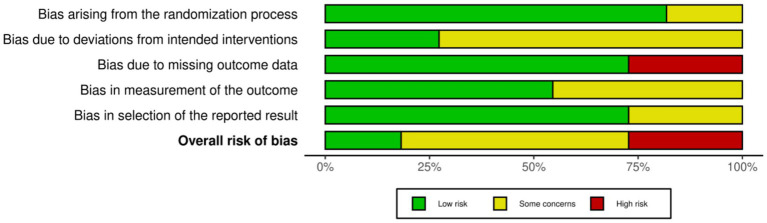
Risk of bias graph: review authors’ judgments about each risk of bias item, presented as percentage of included studies. This graph shows the risk of bias assessment results for each included study across the five domains, according to the Cochrane RoB 2.0 tool. Detailed assessment methods are described in Methods section 2.4, and a detailed narrative of the assessment results is provided in Results section 3.3.

According to the Cochrane RoB 2.0 tool assessment, the overall quality of the 11 included studies was generally good, as shown in Figures 2, 3. Regarding the randomization process (D1), two studies had risks, mainly due to failure to ensure allocation sequence concealment: An et al. (30) explicitly used a “simple coin toss method”; Ho et al. (33) used “cluster randomization” and allocated by “drawing lots,” which also lacked measures to prevent allocation prediction. For deviations from intended interventions (D2), due to the nature of the interventions (e.g., remote APP vs. face-to-face or usual control), eight studies could not objectively blind participants and intervention providers (28, 29, 31, 32, 34–37), and were thus rated as “Some concerns.” Missing outcome data (D3) was the primary source of bias in this assessment. Three studies were objectively rated as “High risk” in this domain: Wang et al. (29) clearly reported a high attrition rate of 19.4% in the exercise group; Wei et al. (37) reported an attrition rate of 22.6%; and Zhang et al. (36) reported a significant differential attrition rate of 17.2% vs. 6.9%. According to Cochrane standards, because these three studies did not report (not reported) using intention-to-treat (ITT) analysis to handle these high and unbalanced missing data, their bias risk was rated as high. Additionally, one study (28) was rated “Some concerns” for not reporting any attrition data at the 6-month follow-up. For bias in measurement of the outcome (D4), five studies were rated “Some concerns” for not reporting whether outcome assessors were blinded (28, 29, 31, 33, 37). For bias in the selection of the reported result (D5), eight studies were rated Low risk (27–29, 31, 33, 35–37). Three studies had “Some concerns” (30, 32, 34), for objective reasons, including not reporting trial registration or explicitly stating retrospective registration. Overall, three studies were rated “Overall High risk” due to objective flaws in D3 (29, 36, 37). Six studies were rated “Some concerns” (28, 30–34). Two studies were rated “Low risk” (27, 35). This result suggests that the findings of this meta-analysis must be interpreted with close attention to potential bias from missing data.

### 3.4 Meta-analysis results

#### 3.4.1 Muscle mass

**Figure 4:**
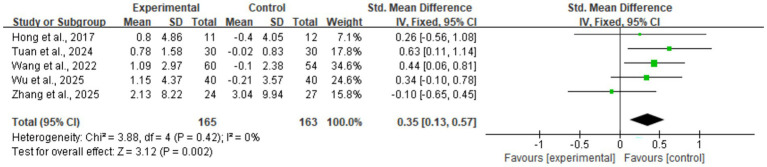
Forest plot of skeletal muscle mass. The meta-analysis results show that, compared to the control group, digital health interventions significantly improved skeletal muscle mass in older adults with sarcopenia. The pooled effect size was calculated using a fixed-effect model (SMD = 0.35, 95% CI: 0.13–0.57, *p* < 0.01), and there was no statistical heterogeneity among studies (*I*^2^ = 0%).

**Figure 5:**
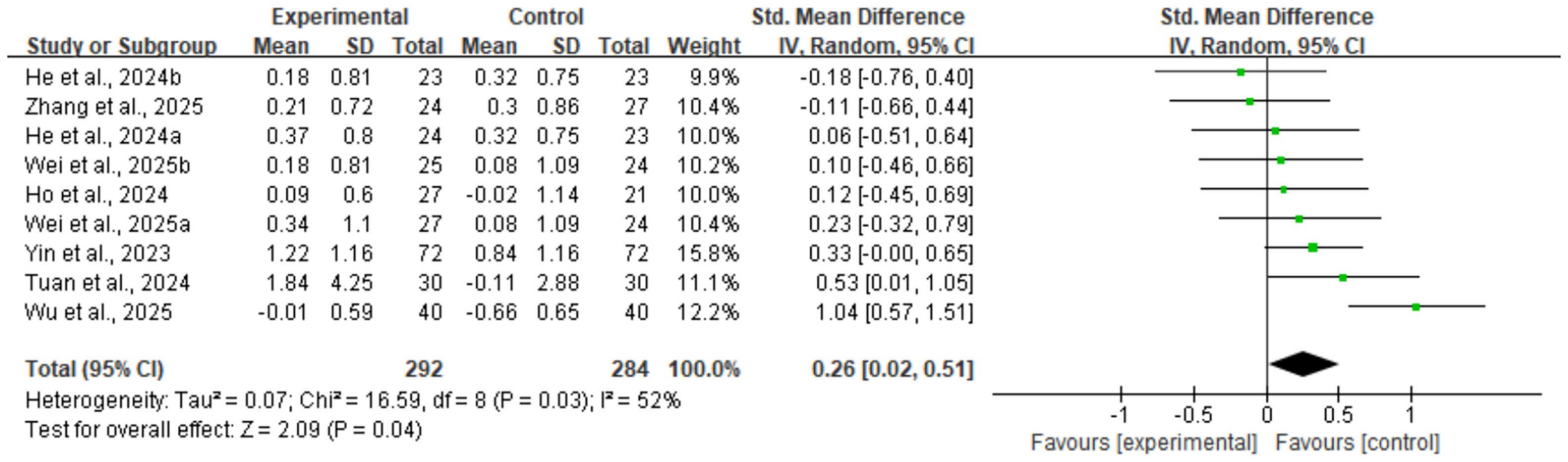
Forest plot of skeletal muscle mass index. The meta-analysis results show that, compared to the control group, digital health interventions significantly improved the skeletal muscle mass index in older adults with sarcopenia. The pooled effect size was calculated using a random-effects model (SMD = 0.26, 95% CI: 0.02–0.51, *p* = 0.04), with moderate heterogeneity among studies (*I*^2^ = 52%).

The meta-analysis results showed that digital health interventions had a positive effect on improving muscle mass in older adults with sarcopenia. The pooled analysis for skeletal muscle mass showed a statistically significant improvement (five studies, *n* = 328; SMD = 0.35, 95% CI: 0.13–0.57, *p* < 0.01), with high consistency among studies (*I*^2^ = 0%) (see Figure 4). Similarly, a significant positive effect was observed for skeletal muscle mass index (nine studies, *n* = 576; SMD = 0.26, 95% CI: 0.02–0.51, *p* = 0.04) (see Figure 5). To explore the moderate heterogeneity (*I*^2^ = 52%) in skeletal muscle mass index, we conducted a sensitivity analysis. The results indicated that Wu et al. (34) was the primary source of heterogeneity; after its removal, heterogeneity was eliminated (*I*^2^ = 0%), and the pooled effect size remained statistically significant (SMD = 0.18, p = 0.04), confirming the robustness of the result. Furthermore, the pre-specified subgroup analyses (Supplementary Figures 1, 2) showed no statistically significant differences in effects between groups, whether grouped by intervention duration (>8 weeks vs. ≤ 8 weeks) or technology interactivity (high vs. low) (*p* > 0.05).

#### 3.4.2 Muscle strength

**Figure 6:**
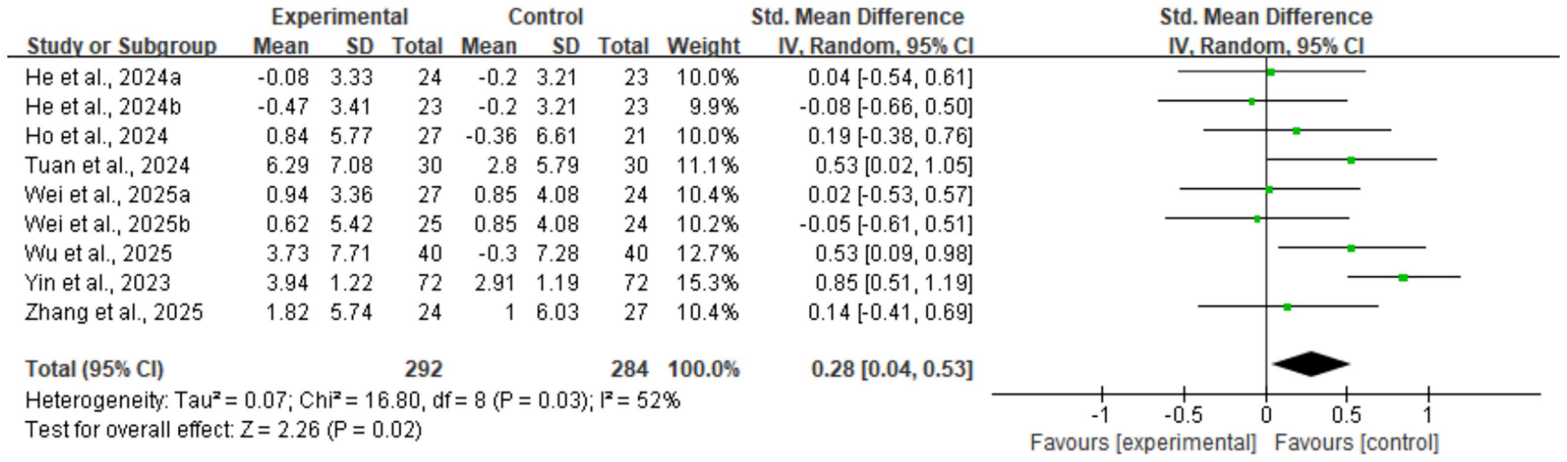
Forest plot of grip strength. The meta-analysis results show that digital health interventions significantly improved the grip strength of older adults with sarcopenia. The pooled effect size was calculated using a random-effects model (SMD = 0.28, 95% CI: 0.04–0.53, *p* = 0.02), with moderate heterogeneity among studies (*I*^2^ = 52%).

Regarding muscle strength, DHIs also led to effective improvements. The meta-analysis for grip strength, a core diagnostic criterion for sarcopenia, confirmed this improvement (nine studies, *n* = 576; SMD = 0.28, 95% CI: 0.04–0.53, *p* = 0.02) (see Figure 6). This analysis had moderate heterogeneity (*I*^2^ = 52%). Sensitivity analysis indicated that Yin et al. (28) was the main source of heterogeneity; its removal eliminated heterogeneity (*I*^2^ = 0%), and the result remained statistically significant (SMD = 0.20, *p* = 0.04), demonstrating robustness. Subsequent subgroup analyses (Supplementary Figures 3, 4) showed that neither intervention duration nor technology interactivity significantly explained the heterogeneity (between-group *p* > 0.05).

#### 3.4.3 Physical function

In contrast to the clear positive effects on muscle mass and strength, the impact of DHIs on physical function appeared to be selective. Specifically, observed improvements included a significant reduction in the time for a fixed-number sit-to-stand test (two studies; SMD = −0.63, *p* = 0.02) (Supplementary Figure 5), suggesting a potential benefit for lower limb power. However, the result for the Timed Up and Go Test (TUGT) (six studies; SMD = 0.32, *p* = 0.05) (Supplementary Figure 6) was at the statistical threshold, showing no significant difference. This improvement did not extend to all physical function indicators. Notably, no statistically significant changes were observed in key mobility metrics, including gait speed over a specified distance (six studies; *p* = 0.73) (Supplementary Figure 7) and walking distance in a specified time (two studies; *p* = 0.40) (Supplementary Figure 8). Furthermore, the number of sit-to-stands in a specified time (two studies; *p* = 0.12) (Supplementary Figure 9) also showed no significant improvement.

#### 3.4.4 Secondary outcomes

Assessment of secondary outcomes also revealed uncertainty. For Quality of Life (QoL), the pooled analysis initially showed a moderate improvement (five studies; SMD = 0.47, *p* = 0.02) (Supplementary Figure 10). However, this result had moderate heterogeneity (*I*^2^ = 57%) and lacked robustness. Sensitivity analysis showed that after excluding He et al. (27), the pooled effect lost statistical significance (SMD = 0.31, *p* = 0.34), suggesting the current evidence is inconclusive. Meanwhile, for Activities of Daily Living (ADL), the pooled analysis showed no significant difference compared to the control group (three studies; SMD = 0.43, *p* = 0.39), and this result was affected by very high heterogeneity (*I*^2^ = 91%) (Supplementary Figure 11). A summary of the meta-analysis results is provided in Table 2.

**Table 2:** Summary of meta-analysis results for all outcomes.

Outcome	Studies (*n*)	Participants (I/C)	SMD (95% CI)	*p*-value	*I* ^2^	Model	Sensitivity analysis
Skeletal muscle mass	5	165/163	0.35 (0.13, 0.57)	<0.01	0%	Fixed	NA
Skeletal muscle mass index	9	292/284	0.26 (0.02, 0.51)	0.04	52%	Random	After excluding Wu et al. (34): SMD = 0.18, *I*^2^ = 0%
Grip strength	9	292/284	0.28 (0.04, 0.53)	0.02	52%	Random	After excluding Yin et al. (28): SMD = 0.20, *I*^2^ = 0%
Walk test (speed)	6	129/124	0.04 (−0.20, 0.29)	0.73	0%	Fixed	NA
Walk Test (distance)	2	50/54	0.33 (−0.43, 1.09)	0.40	73%	Random	NA
Timed up and go test	6	134/133	0.32 (−0.00, 0.65)	0.05	43%	Random	NA
Sit-to-stand (time)	2	67/61	−0.63 (−1.14, −0.12)	0.02	49%	Random	NA
Sit-to-stand (reps)	2	84/81	−0.25 (−0.55, 0.06)	0.12	0%	Fixed	NA
Quality of life	5	115/109	0.47 (0.06, 0.88)	0.02	57%	Random	After excluding He et al. (27): SMD = 0.31, *I*^2^ = 34%
Activities of daily living	3	111/114	0.43 (−0.56, 1.43)	0.39	91%	Random	NA

This table summarizes the pooled standardized mean differences (SMD) for all outcome measures. The results show that digital health interventions led to significant improvements in skeletal muscle mass (SMD = 0.35, *p* < 0.01) and grip strength (SMD = 0.28, *p* = 0.02), but no significant differences were seen in walking speed (*p* = 0.73) or activities of daily living (*p* = 0.39). SMD, standardized mean difference; CI, confidence interval; I/C, intervention group/control group.

### 3.5 Certainty of evidence

Based on the GRADE system, the certainty of evidence for this review ranged from very low to moderate (see Table 3). The certainty of evidence for skeletal muscle mass was moderate (five studies, 328 patients) (29, 32, 34–36). This outcome was downgraded due to the inclusion of high-risk-of-bias studies. The evidence for two core indicators, skeletal muscle mass index (27, 28, 33, 35–37) and grip strength (27, 28, 33–37), was low (nine studies each, 576 patients). Both were downgraded twice: once for high risk of bias and once for moderate heterogeneity. The evidence for physical function indicators was mostly low or very low. Gait speed (six studies, 253 patients) (27, 34–36), TUGT (six studies, 267 patients) (27, 32, 36, 37), and the number of sit-to-stand in prescribed time (29, 36) were all rated as “low” certainty, downgraded for high risk of bias and imprecision. Time of sit-to-stand in prescribed number (two studies, 128 patients) (27, 28) was rated “moderate,” downgraded only for the small number of studies. The evidence for walking distance (two studies, 104 patients) (31, 36) was “very low,” receiving triple downgrades for high risk of bias, high heterogeneity, and serious imprecision. Quality of Life (five studies, 224 patients) (27, 30, 37) was rated “low.” Activities of Daily Living (three studies, 225 patients) (28, 30, 36) was also rated “very low” due to high risk of bias, serious heterogeneity, and imprecision. Notably, as the number of studies for each outcome was less than 10, publication bias could not be assessed via funnel plots, which may affect the overall confidence in the evidence. Given that the evidence for most primary outcomes was rated “moderate” to “very low,” the conclusions of this study regarding the effectiveness of DHIs should be interpreted with caution.

**Table 3:** GRADE evidence quality evaluation.

Outcome	Studies (participants)	Risk of bias	Inconsistency	Indirectness	Imprecision	Publication bias	Quality of evidence
Skeletal muscle mass	5 (328)	Downgraded^a^	Not downgraded	Not downgraded	Not downgraded	Not assessed^e^	⊕ ⊕ ⊕⊝ Moderate
Skeletal muscle mass index	9 (576)	Downgraded^a^	Downgraded^b^	Not downgraded	Not downgraded	Not assessed^e^	⊕ ⊕ ⊝⊝ Low
Grip strength	9 (576)	Downgraded^a^	Downgraded^b^	Not downgraded	Not downgraded	Not assessed^e^	⊕ ⊕ ⊝⊝ Low
Walking test (speed)	6 (253)	Downgraded^a^	Not downgraded	Not downgraded	Downgraded^c^	Not assessed^e^	⊕ ⊕ ⊝⊝ Low
Walking test (distance)	2 (104)	Downgraded^a^	Downgraded^b^	Not downgraded	Seriously downgraded^d^	Not assessed^e^	⊕⊝⊝⊝ Very low
Timed up and go test	6 (267)	Downgraded^a^	Not downgraded	Not downgraded	Downgraded^c^	Not assessed^e^	⊕ ⊕ ⊝⊝ Low
Sit-to-stand test (time)	2 (128)	Not downgraded	Not downgraded	Not downgraded	Downgraded^d^	Not assessed (e)	⊕ ⊕ ⊕⊝ Moderate
Sit-to-stand test (repetitions)	2 (165)	Downgraded^a^	Not downgraded	Not downgraded	Downgraded^c^	Not assessed^e^	⊕ ⊕ ⊝⊝ Low
Quality of life	5 (224)	Downgraded^a^	Downgraded^b^	Not downgraded	Downgraded^c^	Not assessed^e^	⊕ ⊕ ⊝⊝ Low
Activities of daily living	3 (225)	Downgraded^a^	Seriously downgraded^b^	Not downgraded	Downgraded^c^	Not assessed^e^	⊕⊝⊝⊝ Very low

This table summarizes the certainty of evidence for each outcome measure. The primary outcomes (skeletal muscle mass, grip strength) were rated as high to moderate quality evidence, while secondary outcomes (e.g., activities of daily living) were of very low quality.

a Downgraded due to inclusion of studies with high risk of bias.

b *I*^2^ > 50%.

c Confidence interval includes the null value or is close to the null value.

d No. of studies ≤2 and sample size <200.

e No. of included studies <10, publication bias not assessed.

## 4 Discussion

### 4.1 Summary of findings

This systematic review and meta-analysis aimed to comprehensively evaluate the effects of various DHIs on muscle mass, muscle strength, and physical function in older adults with diagnosed sarcopenia, seeking to fill evidence gaps for this specific population and provide clinical guidance. Based on data from 11 RCTs involving 757 patients, our main findings show that DHIs are significantly effective in improving the core indicators of sarcopenia. This was particularly evident for muscle mass, where skeletal muscle mass (five studies, SMD = 0.35, 95% CI: 0.13–0.57) showed a statistically significant improvement with high consistency (*I*^2^ = 0%), and the evidence was rated as moderate certainty. Similarly, grip strength, the core diagnostic criterion for sarcopenia, also showed a significant increase (nine studies, SMD = 0.28, 95% CI: 0.04–0.53), with moderate heterogeneity (*I*^2^ = 52%) and low certainty evidence. Although the observed effect sizes (SMD 0.28–0.35) are in the small-to-moderate range, their significance is important in the specific clinical context of sarcopenia. First, sarcopenia is an age-related progressive muscle disease, placing patients on a trajectory of continuous functional decline and at high risk for adverse outcomes (e.g., falls, disability). In this context, any statistically significant positive change, even if modest, may represent a containment of this decline, which is crucial for preventing disability. However, the intervention’s impact on physical function was selective; while sit-to-stand time significantly improved (two studies, SMD = –0.63, *p* = 0.02), no significant improvements were seen in mobility indicators such as gait speed and walking distance.

Overall, the clinical implication of these findings is that they provide strong evidence-based support for integrating DHIs as a feasible and effective strategy into the comprehensive management of sarcopenia, especially for patient groups with limited access to traditional rehabilitation services.

### 4.2 Comparison with other studies and mechanistic analysis

Our findings are both consistent with and distinct from previous research. In the DHI field, an umbrella review by Longhini et al. (38) focused on the impact of wearables on physical activity. It found that while wearables effectively improved PA in middle-aged adults, the results in older adult subgroups were inconsistent and uncertain. This finding aligns closely with our observation that functional mobility (i.e., walking ability) did not significantly improve, highlighting that translating the benefits of digital interventions into functional mobility improvements in sarcopenic patients remains challenging and selective. Compared to the systematic review by Makizako et al. (14), which focused on healthy older adults, our study’s focus on diagnosed sarcopenia patients reveals slightly lower but more clinically specific effect sizes, possibly reflecting the reality that sarcopenic patients have lower functional baselines and greater difficulty improving. Notably, these interventions can be categorized by technology integration and delivery mode into asynchronous and synchronous models. Most studies used asynchronous models, providing preset content via mobile apps or VR systems and relying on automated feedback or data monitoring; this model is flexible and scalable (34, 37). A few studies used synchronous models, such as real-time one-on-one remote supervised training via video conferencing (32), which provides stronger personalized guidance and social support. These differences in delivery mode, along with the intervention content itself, constitute the complex mechanisms by which DHIs function.

It must be emphasized that this study did not directly assess the physiological or psychological mechanisms of the interventions. Therefore, the following discussion is based primarily on indirect evidence and theoretical inference from prior literature, intended to provide a possible explanatory framework for our observed results rather than empirical conclusions (39). Based on this premise, the potential mechanisms of DHIs may involve multiple levels. First, from a neuromuscular adaptation perspective, resistance training reportedly improves motor unit recruitment and neuromuscular coordination (40). Visual feedback and real-time guidance in DHIs might enhance motor cortex activation and motor learning efficiency (30), while VR may promote neuroplasticity through enhanced sensorimotor integration (41). Second, behavioral change mechanisms likely played a significant role. Instant feedback, goal setting, and gamification elements in DHIs may help enhance patient self-efficacy and intrinsic motivation, thereby improving long-term adherence (42, 43). From a molecular level, regular exercise is known to regulate the mTOR signaling pathway related to muscle protein synthesis and reduce inflammatory factors, although this review did not measure these biomarkers (44). Furthermore, cognitive engagement may be a unique advantage of DHIs. Many interventions (e.g., MR training) require cognitive-motor dual-task processing; this type of training is believed to improve executive function (45). However, our results showed that improvements in physical function were selective, suggesting that the specific method of cognitive engagement and the use of cognitive strategies are critical. First, real-world gait in older adults is rarely a single task but rather a dual task. A study on older adults confirmed that cognitive strategies induced by positive expectancy (i.e., placebo effect) significantly reduced dual-task costs and improved gait performance (46). This suggests that the positive expectancy built by DHIs may be a key factor in enhancing functional outcomes. Second, the effectiveness of cognitive strategy is modulated by attentional focus. Research has shown that the enhancing effect of cognitive intervention on muscle strength only occurs when participants adopt an internal focus of attention; performance was inhibited with an external focus (47). This finding has direct implications for the different DHI types assessed in our review. Therefore, the cognitive design of a DHI may be a key moderating variable, perhaps explaining the selective functional improvements observed in our study. Sociopsychological factors also cannot be ignored; remote supervision and virtual socialization may reduce feelings of social isolation, and social support is linked to sarcopenia improvement (48, 49). Finally, these mechanistic speculations, based mainly on indirect evidence and theory (50), help explain why the effects of DHIs can be comparable to some traditional face-to-face interventions, but they still require future research with corresponding biomarkers and psychometrics for direct validation.

### 4.3 Study limitations

The innovation of this study lies in its first systematic inclusion of evidence on the application of emerging technologies such as artificial intelligence and mixed reality in sarcopenia intervention, and ensuring the homogeneity of the study population based on international diagnostic standards, providing a reference for the effectiveness of digital health interventions. Although this systematic review provides important evidence on the effects of digital health interventions on older adults with sarcopenia, several limitations exist that require cautious interpretation of the results. First, at the study level, the number of included RCTs is relatively limited, the number of studies for some outcome measures (such as walking distance, sit-to-stand test) is insufficient, and some studies have small sample sizes. This limits the statistical power and generalizability of the results. The small sample sizes in some studies may lead to imprecise effect estimates, as reflected in the wide confidence intervals for several outcome measures. The heterogeneity of interventions is another important limitation; the included studies covered various technological forms, from mobile apps and wearable devices to virtual reality and artificial intelligence, with intervention durations ranging from 4 to 12 weeks. While this heterogeneity reflects the diversity of digital health technologies, it also increases the difficulty of determining the optimal intervention protocol. Furthermore, more than half of the studies had concerns regarding the risk of bias from missing outcome data, which might overestimate the true effect of the interventions.

At the research level, issues such as non-uniformity of measurement methods, the inability to assess publication bias using funnel plots, and the fact that studies were mainly from Asian countries, which may limit the generalizability of the results, still exist. In particular, as mentioned in the methods section, this study’s search strategy did not cover gray literature or preprint servers, and coupled with the fact that the number of studies included for each outcome was less than 10, it was impossible to effectively assess publication bias using funnel plots. Therefore, this study may have failed to include some unpublished negative result studies, posing a potential risk of overestimating the true intervention effects. More importantly, this study failed to deeply explore the adherence to and real-world usability of the interventions. Although the included studies reported generally high completion rates, this does not fully represent high adherence to these technologies in real-world, non-clinically supervised environments. In actual implementation, long-term use by older adults is constrained by various complex factors, none of which were fully evaluated in the current studies. Among these, accessibility and digital literacy constitute the primary barriers, as the digital divide may exclude some older adults lacking equipment or skills from the outset. The inadequacy of age-friendly design in devices is another common problem; many technologies are designed with younger users as the target, and their complex interfaces and operational logic undoubtedly create difficulties for older users. Moreover, psychosocial factors should not be ignored, such as the potential resistance of some older adults to using technologies perceived as “assistive” or “monitoring” due to “stigma.” In addition, this study lacks an assessment of the mechanisms of intervention action. None of the included studies reported physiological mechanism parameters such as inflammatory markers or muscle protein synthesis indicators, nor did they assess psychological mediators like self-efficacy or exercise motivation, limiting the understanding of the intrinsic mechanisms by which digital health technologies improve sarcopenia.

### 4.4 Practical implications

Although the evidence from this study is still preliminary, its results can nonetheless offer some guidance for clinical practice. For the specific population focused on older adults with diagnosed sarcopenia, digital health interventions can be considered as a potential supplementary management strategy, based on the high-to-moderate quality evidence showing improvements in muscle mass and muscle strength. This is particularly applicable to patients who have difficulty accessing traditional rehabilitation due to geographical location, mobility issues, or financial burdens. When developing individualized treatment plans, clinicians should prioritize intervention models that have been proven effective, such as comprehensive programs including resistance and balance training (51, 52). They should also strengthen gait training and dynamic balance exercises, based on this study’s findings regarding the selective nature of improvements in physical function. For technology selection, choices can be flexible based on the patient’s digital literacy and preferences. A hybrid model could be adopted, providing necessary face-to-face guidance during the initial phase of the intervention to ensure safety and accuracy.

From a policy and research perspective, the results of this study support the integration of digital health technologies into the prevention and management systems for sarcopenia in older adults. Policymakers should consider establishing standardized guidelines and quality certification, including effective interventions in health insurance coverage, investing in digital infrastructure, and conducting skills training for healthcare professionals. At the same time, future research should focus on addressing existing evidence gaps, including conducting large-sample, multicenter RCTs covering other populations, performing head-to-head comparison studies of different technologies, evaluating long-term effects, and exploring the potential of artificial intelligence in developing personalized protocols. Furthermore, it is necessary to establish the minimum effective dose and optimal combination model for interventions, and to elucidate their mechanisms of action by including biomarkers and psychological scales.

Furthermore, research exploration should transcend existing technological frameworks, focusing on potential technologies with higher levels of integration and intelligence. First, the application of wearable devices should be deepened, expanding from step counting to collecting multi-dimensional data such as gait and balance. Second, it is crucial to use this data to develop predictive algorithms, with the aim of identifying functional decline in advance or predicting fall risk, thereby achieving active prevention. Finally, developing more complex real-time feedback systems is a direction worth exploring, for example, combining artificial intelligence coaches with augmented/virtual reality (AR/VR) technology to dynamically adjust training based on the user’s real-time physiological data, providing a highly personalized and immersive experience. The fusion of these cutting-edge technologies may help older adults with sarcopenia build a closed-loop, intelligent, and efficient management solution.

## 5 Conclusion

This systematic review and meta-analysis, for the first time, evaluated the comprehensive effects of digital health interventions specifically targeting older adults with diagnosed sarcopenia. Based on evidence from 11 randomized controlled trials, these interventions appear to significantly improve patients’ muscle mass and muscle strength, but their effect on improving overall physical function (especially walking ability) is not yet clear, showing some selectivity. The range of digital technologies evaluated in this study was broad, covering everything from basic mobile applications and wearable monitoring devices to more interactive remote video guidance, exergames, virtual reality, and artificial intelligence real-time feedback systems. Overall, technologies capable of providing real-time, interactive, and personalized feedback show good application prospects. Although the number of studies based on emerging technologies such as artificial intelligence (AI) and virtual/mixed reality (VR/MR) was still small in this review, and the evidence remains preliminary, the real-time interaction and personalized feedback features they demonstrate suggest their potential as future management tools. Therefore, digital health interventions, especially those capable of providing personalized guidance and feedback, offer new possibilities for the future of personalized and remote sarcopenia management. From a public health perspective, the findings of this study are positive; considering the inherent scalability and accessibility of digital interventions, they provide a promising supplementary strategy for addressing the increasingly severe challenges of sarcopenia management in the context of global aging.

## Data Availability

The original contributions presented in the study are included in the article/Supplementary material, further inquiries can be directed to the corresponding authors.
