# Supplementary material for: Effects of digital health interventions on muscle mass, muscle strength, and physical function in older adults with sarcopenia: a systematic review and meta-analysis
Source: Front Public Health. 2025 Nov 26;13:1711514. doi: 10.3389/fpubh.2025.1711514 (PMC12689566; doi:10.3389/fpubh.2025.1711514)
Supplement: Supplementary file 1 [file Supplementary_file_1.docx]

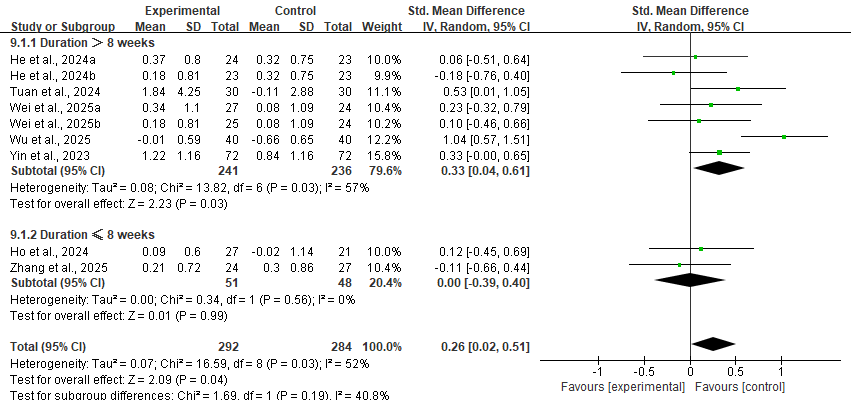


Supplementary Figure 1 Forest plot of skeletal muscle mass index based on subgroup analysis by intervention duration
Caption: Subgroup analysis showed that the subgroup with intervention duration > 8 weeks (P=0.03), compared to the subgroup with duration ≤ 8 weeks (P=0.99), had no statistically significant difference in effect size (between-group P = 0.19), suggesting that intervention duration may not be the main source of heterogeneity.


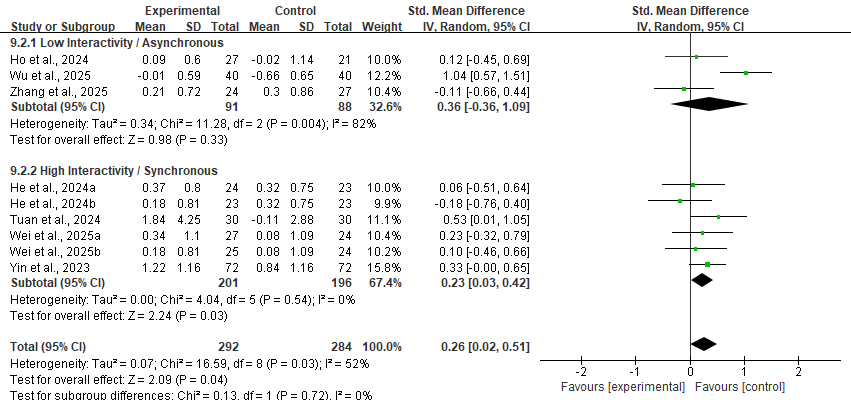


Supplementary Figure 2 Forest plot of skeletal muscle mass index based on subgroup analysis by intervention technology type / interactivity

Caption: Subgroup analysis showed that the low interactivity / asynchronous intervention group (P=0.33), compared to the high interactivity / synchronous intervention group (P=0.03), had no statistically significant difference in effect size (between-group P = 0.72).


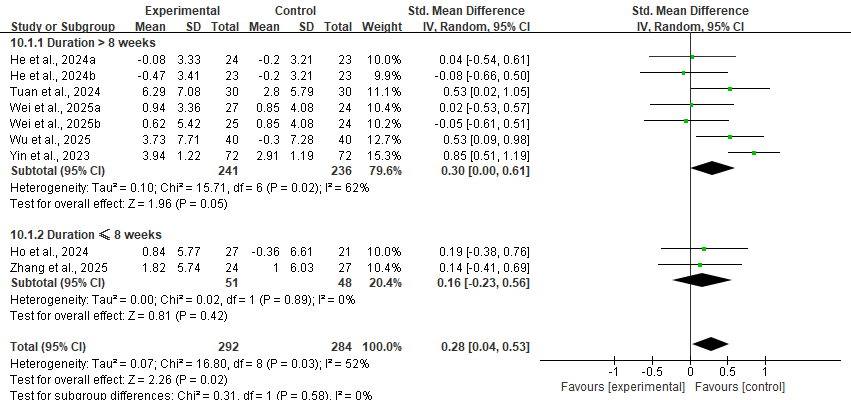


Supplementary Figure 3 Forest plot of grip strength based on subgroup analysis by intervention duration

Caption: Subgroup analysis showed that the subgroup with intervention duration > 8 weeks (P=0.05), compared to the subgroup with duration ≤ 8 weeks (P=0.42), had no statistically significant difference in effect size (between-group P = 0.58).


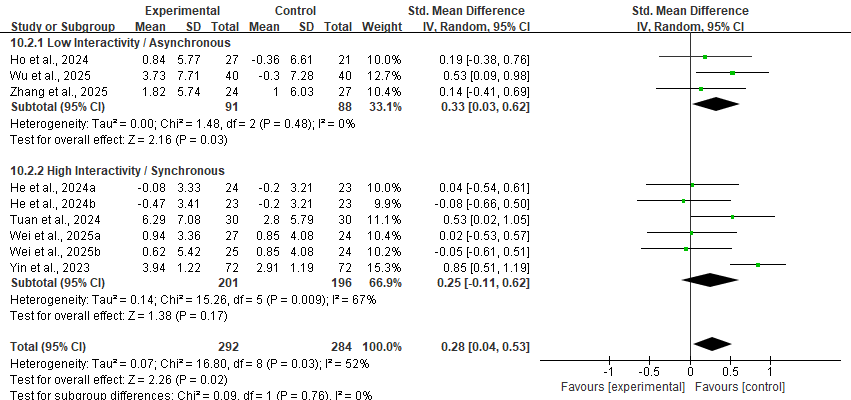


Supplementary Figure 4 Forest plot of grip strength based on subgroup analysis by intervention technology type / interactivity

Caption: Subgroup analysis showed that the low interactivity / asynchronous intervention group (P=0.03), compared to the high interactivity / synchronous intervention group (P=0.17), had no statistically significant difference in effect size (between-group P = 0.76).


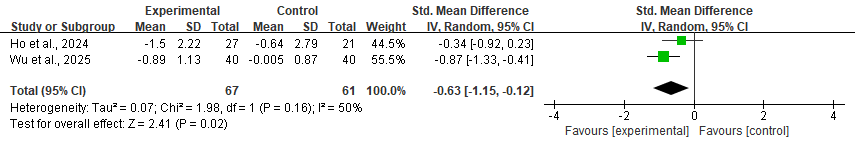


Supplementary Figure 5 Forest plot of time for fixed-number sit-to-stand test

Caption: The pooled analysis showed that digital health interventions significantly shortened sit-to-stand time (SMD=-0.63, 95% CI: -1.15, -0.12, P=0.02; random-effects model), with moderate heterogeneity among studies (I²=50%).


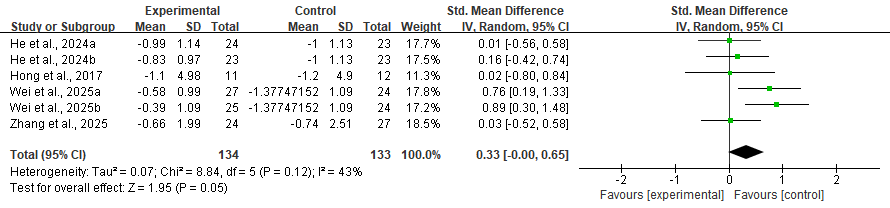

Supplementary Figure 6 Forest plot of timed up and go test

Caption: The pooled analysis showed that digital health interventions had no significant effect on TUGT (SMD=0.32, 95% CI: -0.00-0.65, P=0.05; random-effects model), with the result at the statistical threshold and moderate heterogeneity among studies (I²=43%).

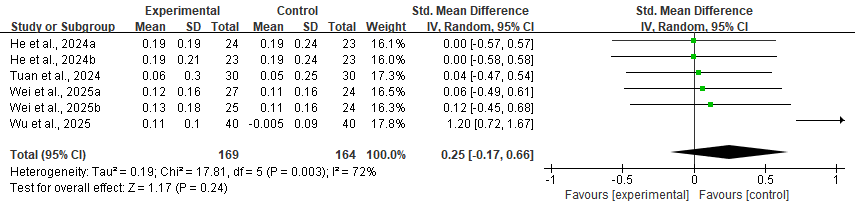


Supplementary Figure 7 Forest plot of walking speed for specified distance

Caption: The pooled analysis showed that digital health interventions had no significant improvement on walking speed (SMD=0.25, 95% CI: -0.17, 0.66, P=0.24; random-effects model), with high heterogeneity among studies (I²=72%).


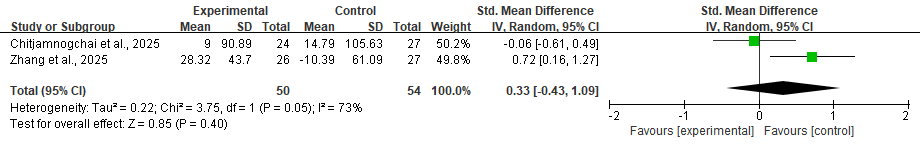


Supplementary Figure 8 Forest plot of walking distance in specified time

Caption: The pooled analysis showed that digital health interventions had no significant improvement on walking distance (SMD=0.33, 95% CI: -0.43, 1.09, P=0.40; random-effects model), with high heterogeneity among studies (I²=73%).


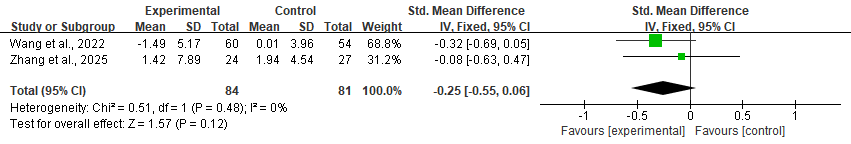


Supplementary Figure 9 Forest plot of number of sit-to-stands in specified time

Caption: The pooled analysis showed that digital health interventions had no significant improvement on sit-to-stand repetitions (SMD=-0.25, 95% CI: -0.55, 0.06, P=0.12; fixed-effect model), with no heterogeneity among studies (I²=0%).


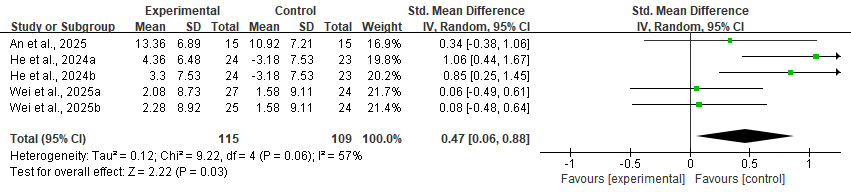


Supplementary Figure 10 Forest plot of quality of life

Caption: The pooled analysis showed that digital health interventions had a significant improvement on quality of life (SMD=0.47, 95% CI: 0.06, 0.88, P=0.03; random-effects model), with moderate heterogeneity among studies (I²=57%).


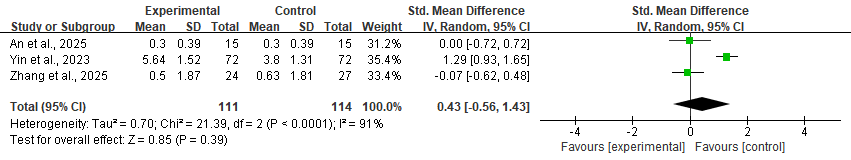


Supplementary Figure 11 Forest plot of activities of daily living

Caption: The pooled analysis showed that digital health interventions had no significant improvement on activities of daily living (SMD=0.43, 95% CI: -0.56, 1.43, P=0.39; random-effects model), with very high heterogeneity among studies (I²=91%).

Supplementary Text 1 Search Strategies

1.PubMed：48 studies

search strategy:

#1 ("sarcopenia"[MeSH Terms])

#2 (Sarcopen*[Title/Abstract] OR "muscle wasting"[Title/Abstract] OR "muscle atrophy"[Title/Abstract] OR "muscle loss"[Title/Abstract])

#3 #1 OR #2

#4 ("Aged"[MeSH Terms])

#5 ("older people*"[Title/Abstract] OR "old age*"[Title/Abstract] OR adult*[Title/Abstract] OR "Aged, 60 and over"[Title/Abstract] OR "older age*"[Title/Abstract] OR "old people*"[Title/Abstract] OR geriatric*[Title/Abstract] OR "older adult*"[Title/Abstract] OR Senior*[Title/Abstract] OR Elderly[Title/Abstract])

#6 #4 OR #5

#7 (telemedicine[Title/Abstract] OR telecommunication*[Title/Abstract] OR Telerehabilitation[Title/Abstract] OR telecare[Title/Abstract] OR "Digital Technolog*"[Title/Abstract] OR "digital health"[Title/Abstract] OR "digital tool"[Title/Abstract] OR "digital care"[Title/Abstract] OR "mobile app*"[Title/Abstract] OR mobileapp*[Title/Abstract] OR ehealth[Title/Abstract] OR "e-health"[Title/Abstract] OR mhealth[Title/Abstract] OR "mobile health"[Title/Abstract] OR "online health"[Title/Abstract] OR "Computers, Handheld"[Title/Abstract] OR "Reminder Systems"[Title/Abstract] OR smartphone*[Title/Abstract] OR "web-based"[Title/Abstract] OR "electronic monitoring"[Title/Abstract] OR "reminder device*"[Title/Abstract] OR "reminder system*"[Title/Abstract] OR "helping hand"[Title/Abstract] OR "Internet-Based Intervention"[Title/Abstract] OR "mobile phone*"[Title/Abstract] OR "cd-rom software"[Title/Abstract] OR "internet website*"[Title/Abstract] OR "e-mail contact*"[Title/Abstract] OR "sms based system*"[Title/Abstract] OR "telemedicine platform*"[Title/Abstract] OR telehealth[Title/Abstract] OR "eHealth intervention*"[Title/Abstract] OR "monitoring device*"[Title/Abstract] OR "mobile application*"[Title/Abstract] OR "digital assistant*"[Title/Abstract] OR "Artificial Intelligence"[Title/Abstract] OR IoT[Title/Abstract] OR chat-bot[Title/Abstract] OR chatbot[Title/Abstract] OR "health website*"[Title/Abstract] OR "Virtual Reality"[Title/Abstract] OR metaverse[Title/Abstract] OR Exergaming[Title/Abstract] OR esports[Title/Abstract] OR e-sports[Title/Abstract] OR "Wearable Electronic Devices"[Title/Abstract] OR wearable[Title/Abstract] OR smartwatch[Title/Abstract] OR "smart watch"[Title/Abstract])

#8 ("grip strength"[Title/Abstract] OR "hand strength"[Title/Abstract] OR "handgrip strength"[Title/Abstract] OR "grip force"[Title/Abstract] OR "muscle strength"[Title/Abstract] OR "timed up and go"[Title/Abstract] OR TUG[Title/Abstract] OR TUGT[Title/Abstract] OR "6-minute walk*"[Title/Abstract] OR "six-minute walk*"[Title/Abstract] OR 6MWT[Title/Abstract] OR "6MWD"[Title/Abstract] OR "walk* test"[Title/Abstract] OR "walk* distance"[Title/Abstract] OR "muscle mass"[Title/Abstract] OR "skeletal muscle mass"[Title/Abstract] OR "appendicular muscle mass"[Title/Abstract] OR ASM[Title/Abstract] OR SMI[Title/Abstract] OR "skeletal muscle index"[Title/Abstract] OR "lean mass"[Title/Abstract] OR IADL[Title/Abstract] OR "instrumental activities of daily living"[Title/Abstract] OR "activities of daily living"[Title/Abstract] OR ADL[Title/Abstract] OR "quality of life"[Title/Abstract] OR QOL[Title/Abstract] OR SF-36[Title/Abstract] OR SF-12[Title/Abstract] OR EQ-5D[Title/Abstract] OR "EuroQol"[Title/Abstract] OR "life quality"[Title/Abstract] OR "health-related quality"[Title/Abstract] OR HRQOL[Title/Abstract])

#9 ("randomized controlled trial"[Publication Type] OR "randomized"[Title/Abstract] OR "placebo"[Title/Abstract])

#10 #3 AND #6 AND #7 AND #8 AND #9

2.Web of Science：81 studies

search strategy:

#1 TS=(sarcopenia OR sarcopenia OR muscle wasting OR muscle atrophy OR muscle loss)

#2 TS=( older people OR adult OR old age OR Aged, 60 and over OR Aged OR older age OR old people OR geriatric OR older adult OR Senior OR Elderly)

#3 TS=(telemedicine OR telecommunication OR Telerehabilitation OR telecare OR "Digital Technolog OR digital health OR digital tool OR digital care OR mobile app OR mobileapp OR ehealth OR e-health OR mhealth OR mobile health OR online health OR Computers, Handheld OR Reminder Systems OR smartphone OR web-based OR electronic monitoring OR reminder device OR reminder system OR helping hand OR Internet-Based Intervention OR mobile phone OR cd-rom software OR internet website OR e-mail contact OR sms based system OR telemedicine platform OR telehealth OR eHealth intervention OR monitoring device OR mobile application OR digital assistant OR Artificial Intelligence OR IoT OR chat-bot OR chatbot OR health website OR Virtual Reality OR metaverse OR Exergaming OR esports OR e-sports OR Wearable Electronic Devices OR wearable OR smartwatch OR smart watch)

#4 TS=(grip strength OR hand strength OR handgrip strength OR grip force OR muscle strength OR timed up and go OR TUG OR TUGT OR 6-minute walk OR six-minute walk OR 6MWT OR 6MWD OR walk test OR walk distance OR muscle mass OR skeletal muscle mass OR appendicular muscle mass OR ASM OR SMI OR skeletal muscle index OR lean mass OR IADL OR instrumental activities of daily living OR activities of daily living OR ADL OR quality of life OR QOL OR SF-36 OR SF-12 OR EQ-5D OR EuroQol OR life quality OR health-related quality OR HRQOL)

#5 TS=(randomized controlled trial OR randomized OR placebo)

#6 #1 AND #2 AND #3 AND #4 AND #5

3.Scopus：94 studies

search strategy:

#1 TITLE-ABS-KEY=(sarcopenia OR sarcopenia OR muscle wasting OR muscle atrophy OR muscle loss)

#2 TITLE-ABS-KEY=(older people OR adult OR old age OR Aged, 60 and over OR Aged OR older age OR old people OR geriatric OR older adult OR Senior OR Elderly)

#3 TITLE-ABS-KEY=(telemedicine OR telecommunication OR Telerehabilitation OR telecare OR Digital Technolog OR digital health OR digital tool OR digital care OR mobile app OR mobileapp OR ehealth OR e-health OR mhealth OR mobile health OR online health OR Computers, Handheld OR Reminder Systems OR smartphone OR web-based OR electronic monitoring OR reminder device OR reminder system OR helping hand OR Internet-Based Intervention OR mobile phone OR cd-rom software OR internet website OR e-mail contact OR sms based system OR telemedicine platform OR telehealth OR eHealth intervention OR monitoring device OR mobile application OR digital assistant OR Artificial Intelligence OR IoT OR chat-bot OR chatbot OR health website OR Virtual Reality OR metaverse OR Exergaming OR esports OR e-sports OR Wearable Electronic Devices OR wearable OR smartwatch OR smart watch)

#4 TITLE-ABS-KEY=(grip strength OR hand strength OR handgrip strength OR grip force OR muscle strength OR timed up and go OR TUG OR TUGT OR 6-minute walk OR six-minute walk OR 6MWT OR 6MWD OR walk test OR walk distance OR muscle mass OR skeletal muscle mass OR appendicular muscle mass OR ASM OR SMI OR skeletal muscle index OR lean mass OR IADL OR instrumental activities of daily living OR activities of daily living OR ADL OR quality of life OR QOL OR SF-36 OR SF-12 OR EQ-5D OR EuroQol OR life quality OR health-related quality OR HRQOL)

#5 TITLE-ABS-KEY=(randomized controlled trial OR randomized OR placebo)

#6 #1 AND #2 AND #3 AND #4 AND #5

4.Embase：33studies

search strategy:

#1 ti,ab,kw=(sarcopenia OR sarcopenia OR muscle wasting OR muscle atrophy OR muscle loss)

#2 ti,ab,kw=(older people OR adult OR old age OR Aged, 60 and over OR Aged OR older age OR old people OR geriatric OR older adult OR Senior OR Elderly)

#3 ti,ab,kw=(telemedicine OR telecommunication OR Telerehabilitation OR telecare OR Digital Technolog OR digital health OR digital tool OR digital care OR mobile app OR mobileapp OR ehealth OR e-health OR mhealth OR mobile health OR online health OR Computers, Handheld OR Reminder Systems OR smartphone OR web-based OR electronic monitoring OR reminder device OR reminder system OR helping hand OR Internet-Based Intervention OR mobile phone OR cd-rom software OR internet website OR e-mail contact OR sms based system OR telemedicine platform OR telehealth OR eHealth intervention OR monitoring device OR mobile application OR digital assistant OR Artificial Intelligence OR IoT OR chat-bot OR chatbot OR health website OR Virtual Reality OR metaverse OR Exergaming OR esports OR e-sports OR Wearable Electronic Devices OR wearable OR smartwatch OR smart watch)

#4 ti,ab,kw=(grip strength OR hand strength OR handgrip strength OR grip force OR muscle strength OR timed up and go OR TUG OR TUGT OR 6-minute walk OR six-minute walk OR 6MWT OR 6MWD OR walk test OR walk distance OR muscle mass OR skeletal muscle mass OR appendicular muscle mass OR ASM OR SMI OR skeletal muscle index OR lean mass OR IADL OR instrumental activities of daily living OR activities of daily living OR ADL OR quality of life OR QOL OR SF-36 OR SF-12 OR EQ-5D OR EuroQol OR life quality OR health-related quality OR HRQOL)

#5 ti,ab,kw=(randomized controlled trial OR randomized OR placebo)

#6 #1 AND #2 AND #3 AND #4 AND #5

5.CINAHL：3 studies

search strategy:

#1 XB=(sarcopenia OR sarcopenia OR muscle wasting OR muscle atrophy OR muscle loss)

#2 XB=( older people OR adult OR old age OR Aged, 60 and over OR Aged OR older age OR old people OR geriatric OR older adult OR Senior OR Elderly)

#3 XB=(telemedicine OR telecommunication OR Telerehabilitation OR telecare OR "Digital Technolog OR digital health OR digital tool OR digital care OR mobile app OR mobileapp OR ehealth OR e-health OR mhealth OR mobile health OR online health OR Computers, Handheld OR Reminder Systems OR smartphone OR web-based OR electronic monitoring OR reminder device OR reminder system OR helping hand OR Internet-Based Intervention OR mobile phone OR cd-rom software OR internet website OR e-mail contact OR sms based system OR telemedicine platform OR telehealth OR eHealth intervention OR monitoring device OR mobile application OR digital assistant OR Artificial Intelligence OR IoT OR chat-bot OR chatbot OR health website OR Virtual Reality OR metaverse OR Exergaming OR esports OR e-sports OR Wearable Electronic Devices OR wearable OR smartwatch OR smart watch)

#4 XB=(grip strength OR hand strength OR handgrip strength OR grip force OR muscle strength OR timed up and go OR TUG OR TUGT OR 6-minute walk OR six-minute walk OR 6MWT OR 6MWD OR walk test OR walk distance OR muscle mass OR skeletal muscle mass OR appendicular muscle mass OR ASM OR SMI OR skeletal muscle index OR lean mass OR IADL OR instrumental activities of daily living OR activities of daily living OR ADL OR quality of life OR QOL OR SF-36 OR SF-12 OR EQ-5D OR EuroQol OR life quality OR health-related quality OR HRQOL)

#5 XB=(randomized controlled trial OR randomized OR placebo)

#6 #1 AND #2 AND #3 AND #4 AND #5

6.Cochrane Library：93 studies

search strategy:

#1 ti,ab,kw=(sarcopenia OR sarcopenia OR muscle wasting OR muscle atrophy OR muscle loss)

#2 ti,ab,kw=(older people OR adult OR old age OR Aged, 60 and over OR Aged OR older age OR old people OR geriatric OR older adult OR Senior OR Elderly)

#3 ti,ab,kw=(telemedicine OR telecommunication OR Telerehabilitation OR telecare OR Digital Technolog OR digital health OR digital tool OR digital care OR mobile app OR mobileapp OR ehealth OR e-health OR mhealth OR mobile health OR online health OR Computers, Handheld OR Reminder Systems OR smartphone OR web-based OR electronic monitoring OR reminder device OR reminder system OR helping hand OR Internet-Based Intervention OR mobile phone OR cd-rom software OR internet website OR e-mail contact OR sms based system OR telemedicine platform OR telehealth OR eHealth intervention OR monitoring device OR mobile application OR digital assistant OR Artificial Intelligence OR IoT OR chat-bot OR chatbot OR health website OR Virtual Reality OR metaverse OR Exergaming OR esports OR e-sports OR Wearable Electronic Devices OR wearable OR smartwatch OR smart watch)

#4 ti,ab,kw=(grip strength OR hand strength OR handgrip strength OR grip force OR muscle strength OR timed up and go OR TUG OR TUGT OR 6-minute walk OR six-minute walk OR 6MWT OR 6MWD OR walk test OR walk distance OR muscle mass OR skeletal muscle mass OR appendicular muscle mass OR ASM OR SMI OR skeletal muscle index OR lean mass OR IADL OR instrumental activities of daily living OR activities of daily living OR ADL OR quality of life OR QOL OR SF-36 OR SF-12 OR EQ-5D OR EuroQol OR life quality OR health-related quality OR HRQOL)

#5 ti,ab,kw=(randomized controlled trial OR randomized OR placebo)

#6 #1 AND #2 AND #3 AND #4 AND #5

7.ClinicalTrials.gov：214 studies

search strategy:

Condition or disease: Sarcopenia OR "muscle wasting" OR "muscle atrophy" OR "muscle loss"

Intervention/treatment: telemedicine OR telecommunication OR Telerehabilitation OR telecare OR "Digital Technology" OR "digital health" OR "digital tool" OR "digital care" OR "mobile app" OR mobileapp OR ehealth OR "e-health" OR mhealth OR "mobile health" OR "online health" OR "Computers, Handheld" OR "Reminder Systems" OR smartphone OR "web-based" OR "electronic monitoring" OR "reminder device" OR "reminder system" OR "helping hand" OR "Internet-Based Intervention" OR "mobile phone" OR "cd-rom software" OR "internet website" OR "e-mail contact" OR "sms based system" OR "telemedicine platform" OR telehealth OR "eHealth intervention" OR "monitoring device" OR "mobile application" OR "digital assistant" OR "Artificial Intelligence" OR IoT OR "chat-bot" OR chatbot OR "health website" OR "Virtual Reality" OR metaverse OR Exergaming OR esports OR "e-sports" OR "Wearable Electronic Devices" OR wearable OR smartwatch OR "smart watch"

Other terms: "older people" OR "old age" OR "Aged, 60 and over" OR "older age" OR "old people" OR geriatric OR "older adult" OR Senior OR Elderly) AND ("grip strength" OR "hand strength" OR "handgrip strength" OR "grip force" OR "muscle strength" OR "timed up and go" OR TUG OR TUGT OR "6-minute walk" OR "six-minute walk" OR 6MWT OR "6MWD" OR "walk test" OR "walk distance" OR "muscle mass" OR "skeletal muscle mass" OR "appendicular muscle mass" OR ASM OR SMI OR "skeletal muscle index" OR "lean mass" OR IADL OR "instrumental activities of daily living" OR "activities of daily living" OR ADL OR "quality of life" OR QOL OR SF-36 OR SF-12 OR EQ-5D OR EuroQol OR "life quality" OR "health-related quality" OR HRQOL)

8.CNKI：112 studies

search strategy:

#1 老年人 + 老年人口 + 老年人群 + 60岁及以上老年人

#2 肌少症 + 肌少症患者

#3 干预 + 干预前后 + 干预研究 + 干预效果

#4 #1 AND #2 AND #3


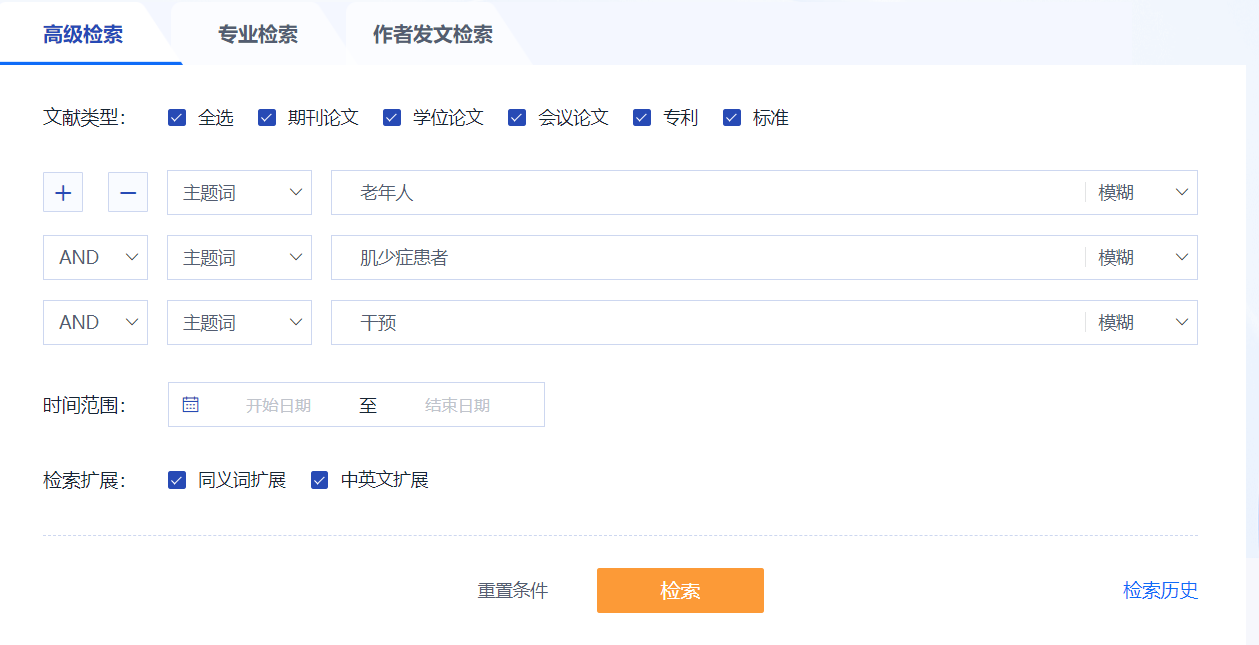


9.Wan fang：310 studies

search strategy:

#1 老年人 + 老年人口 + 老年人群 + 60岁及以上老年人

#2 肌少症 + 肌少症患者

#3 干预 + 干预前后 + 干预研究 + 干预效果

#4 #1 AND #2 AND #3


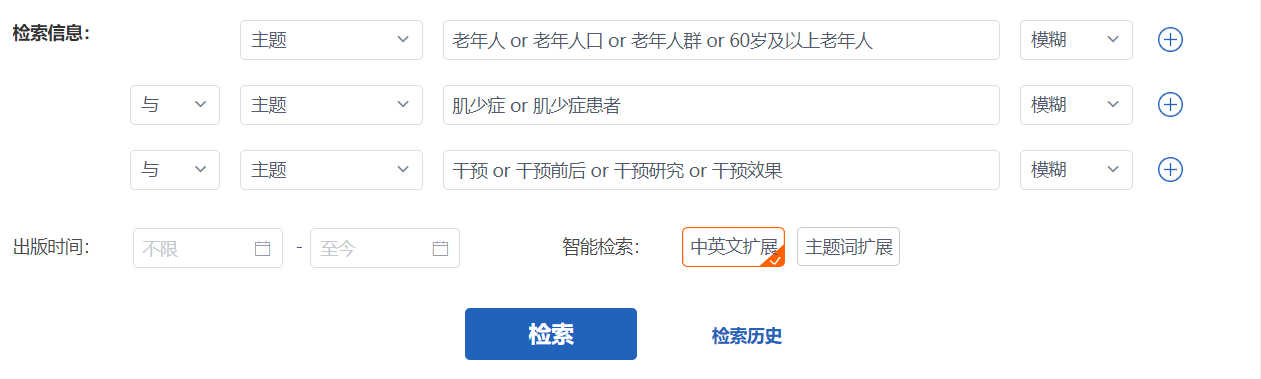


10.VIP：33 studies

search strategy:

#1 老年人 or 老年人口 or 老年人群 or 60岁及以上老年人

#2 肌少症 or 肌少症患者

#3 干预 or 干预前后 or 干预研究 or 干预效果

#4 #1 AND #2 AND #3


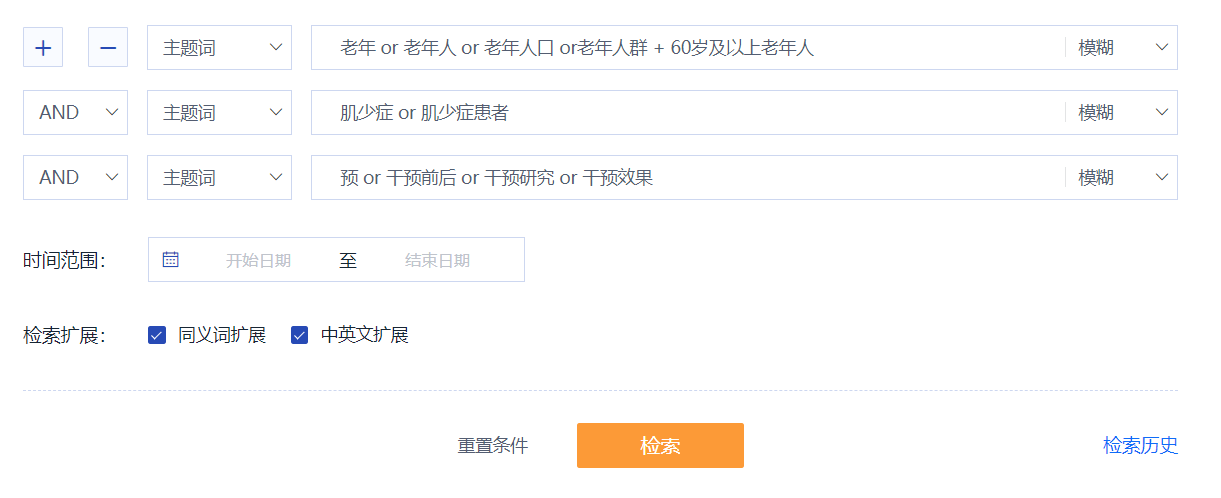


Supplementary Table 1 List of Excluded Studies and Reasons for Exclusion

| **Exclusion Reason, Reference, Title** | **Reasons for Exclusion** |
| --- | --- |
| **Reason 1: Study design mismatch (n=12)** |  |
| Nipp et al., 2018,  Sarcopenia Is Associated with Quality of Life and Depression in Patients with Advanced Cancer | This is an observational study (cross-sectional / cohort), aimed at exploring correlations, not a randomized controlled trial (RCT) |
| Abedi et al., 2025,  Multimodal sensor dataset for monitoring older adults post lower limb fractures in community settings | This is an observational data collection study, not a randomized controlled trial (RCT) |
| Ruiz-Cárdenas et al., 2023,  Concurrent and discriminant validity and reliability of an Android App to assess time, velocity and power during sit-to-stand test in community-dwelling older adults | This is a “Validation Study”, aimed at evaluating the accuracy of an APP,  not an interventional RCT |
| Seinsche et al., 2023,  A Newly Developed Exergame-Based Telerehabilitation System for Older Adults: Usability and Technology Acceptance Study | This is a “Usability and Technology Acceptance Study”, not an efficacy RCT |
| Peng et al., 2020,  Novel Mat Exergaming to Improve the Physical Performance, Cognitive Function, and Dual-Task Walking and Decrease the Fall Risk of Community-Dwelling Older Adults | This is a “non-randomized controlled intervention study” |
| Du et al., 2025,  Development of a visualized risk prediction system for sarcopenia in older adults using machine learning: a cohort study based on CHARLS | This is a retrospective cohort study, not a randomized controlled trial (RCT). |
| Rodrigues et al., 2024,  Mapping sedentary behaviour (MAPS-B) in winter and spring using wearable sensors, indoor positioning systems, and diaries in older adults who are pre-frail and frail: A feasibility longitudinal study | This is a mixed-methods longitudinal observational study |
| Parvaneh et al., 2017,  Postural transitions during activities of daily living could identify frailty status – Application of wearable technology to identify frailty during unsupervised condition. | This is an observational study (cross-sectional / cohort), aimed at exploring correlations, not a randomized controlled trial (RCT) |
| Sarkar et al., 2022,  Enhanced Telehealth Home-Monitoring Intervention for Vulnerable and Frail Patients after Cardiac Surgery (THE-FACS Pilot Intervention Study) | This is a prospective, non-randomized case-control pilot study |
| Petersen et al., 2021,  Remote Rehabilitation: A Field-Based Feasibility Study of an mHealth Resistance Exercise Band | This is a single-arm pilot study conducted on 7 elderly people over 65 years old |
| Upatising et al., 2013,  Effects of home telemonitoring on transitions between frailty states and death for older adults: a randomized controlled trial | This study is a secondary analysis of data from the “Tele-ERA study” |
| Bertschi et al., 2022,  Impact of sarcopenia on daily functioning: a cross-sectional study among older inpatients | This is a “Cross-sectional study”, aimed at exploring “correlation” |
| **Reason 2: Study population mismatch (n=19)** |  |
| Bailey et al., 2024,  The frail-LESS (LEss sitting and sarcopeniain frail older adults) remote interventionto improve sarcopenia and maintainindependent living via reductionsin sedentary behaviour: fndings from arandomised controlled feasibility trial | The included population was “Frail”,  not the “diagnosed sarcopenia” patients required by this study |
| Pan et al., 2025,  Effect of short-term exercise with different programs on prevention of sarcopenia in postmenopausal women: A Quasi-Randomized Controlled Trial | The included population was “women at risk for sarcopenia”, not “diagnosed sarcopenia” patients |
| Chan et al., 2017,  Integrated care for geriatric frailty and sarcopenia: a randomized control trial | The included population was “Frail” elderly people (based on CHS_PCF score), not “diagnosed sarcopenia” patients |
| Lu et al., 2021,  Effects of multi-domain lifestyle interventions on sarcopenia measures and blood biomarkers: secondary analysis of a randomized controlled trial of community-dwelling pre-frail and frail older adults | The included population was “community pre-frail and frail elderly people”, not “diagnosed sarcopenia” patients |
| Landi et al., 2017,  The Sarcopenia and Physical fRailty IN older people: multi-componenT Treatment strategies (SPRINTT) randomized controlled trial: design and methods | The included population was “frail and sarcopenic” elderly people with “mobility impairment”, not the “diagnosed sarcopenia” patients required by this study. |
| Liu et al., 2014,  The impact of sarcopenia on a physical activity intervention: the Lifestyle Interventions and Independence for Elders Pilot Study (LIFE-P) | The included population was elderly people “at risk of mobility impairment”,  not “diagnosed sarcopenia” patients |
| Morone et al., 2016,  Wii Fit is effective in women with bone loss condition associated with balance disorders: a randomized controlled trial | The intervention object of the study was women with bone loss related to balance disorders |
| Chun et al., 2024,  Application study of remote diet and exercise intervention in elderly patients with type 2 diabetes and sarcopenia | The included population was “type 2 diabetes with sarcopenia” elderly patients,  not “diagnosed sarcopenia” patients |
| Wei et al., 2021,  Effects of comprehensive intervention measures in community hospitals on muscle mass and physical function in elderly patients with possible sarcopenia | The included population was “possible sarcopenia” elderly patients, not “diagnosed sarcopenia” patients |
| Ming et al., 2021,  Application effect of home-hospital linkage management model in elderly patients with chronic obstructive pulmonary disease and sarcopenia | The included population was “chronic obstructive pulmonary disease with sarcopenia” elderly patients, not “diagnosed sarcopenia” patients |
| Kannan et al., 2024,  Gaming-Based Tele-Exercise Program to Improve Physical Function in Frail Older Adults: Feasibility Randomized Controlled Trial | The included population was "frail" elderly people, not the "diagnosed sarcopenia" patients required by this study |
| Ji et al., 2025,  Effect of Exercise and Nutrition Intervention for Older Adults with Impaired Physical Function with Preserved Muscle Mass (Functional Sarcopenia): A Randomized Controlled Trial | The included population was elderly people with “impaired physical function but preserved muscle mass”, not the “diagnosed sarcopenia” patients required by this study |
| Warmerdam et al., 2025,  Recovery of Patient-Reported Outcome Measures vs Gait Parameters Obtained by Instrumented Insoles After Tibial and Malleolar Fractures: Prospective Longitudinal Observational Study | The included population was post-operative patients with tibial and ankle fractures, not the “diagnosed sarcopenia” patients required by this study |
| Chen et al., 2017,  Effects of Different Types of Exercise on Body Composition,Muscle Strength, and IGF-1 in the Elderly with Sarcopenic Obesity | The study population includes multiple diseases |
| Wu et al ., 2022,  Clinical Effectiveness of Home‐Based Telerehabilitation Program for Geriatric Hip Fracture Following Total Hip Replacement | The included population was post-operative patients with hip fractures,  not the “diagnosed sarcopenia” patients required by this study, and it is also a non-randomized controlled study |
| Yadav et al ., 2021,  Identifying Opportunities, and Motivation to Enhance Capabilities, Influencing the Development of a Personalized Digital Health Hub Model of Care for Hip Fractures: Mixed Methods Exploratory Study | The included population was patients at risk for sarcopenia, not the “diagnosed sarcopenia” patients required by this study |
| Elena et al., 2020,  Effects of a two-year health-enhancing physical activity program on skeletal muscle protein profiles in people with rheumatoid arthritis | The included population was patients with rheumatoid arthritis lacking physical activity, not the “diagnosed sarcopenia” patients required by this study |
| Nhat et al., 2024,  Clinical evaluation of AI-assisted muscle ultrasound for monitoring muscle wasting in ICU patients | The included population was patients with icu muscle atrophy, not the "diagnosed sarcopenia" patients required by this study |
| Daly et al., 2021,  Feasibility, Usability, and Enjoyment of a Home-Based ExerciseProgram Delivered via an Exercise App for Musculoskeletal Healthin Community-Dwelling Older Adults: Short-term Prospective PilotStudy | The study mainly focuses on musculoskeletal health |
| **Reason 3: Intervention mismatch (n=8)** |  |
| He et al., 2024,  Self-determined sequence exercise program for elderly with sarcopenia: A Randomized controlled trial with clinical assistance from explainable artificial intelligence | The intervention is traditional, self-selected order exercise items (such as strength training, Yi Jin Jing). Artificial intelligence (AI) is only used for prediction and analysis of results, not delivered to patients as a core digital health intervention means (such as APP, remote guidance, or VR). |
| Yamada et al., 2015,  Mail-Based Intervention for Sarcopenia Prevention Increased Anabolic Hormone and Skeletal Muscle Mass in Community-Dwelling Japanese Older Adults: The INE (Intervention by Nutrition and Exercise) Study | The intervention was a traditional “mail-based intervention” and “pedometer” walking program, which does not meet the definition of “Digital Health Intervention” (DHI) for this study |
| Yuenyongchaiwat & Akekawatchai, 2022,  Beneficial effects of walking-based home programfor improving cardio-respiratory performanceand physical activity in sarcopenic older people:a randomized controlled | The intervention was traditional home-based walking and elastic band training. Although a “pedometer” was used, it is a non-smart, non-connected monitoring tool and does not meet  the definition of "Digital Health Intervention" for this study |
| Wei et al., 2022,  Hybrid Exercise Program for Sarcopenia in Older Adults:The Effectiveness of Explainable Artificial Intelligence-BasedClinical Assistance in Assessing Skeletal Muscle Area | The intervention was traditional face-to-face exercise (Yi Jin Jing and resistance training) |
| Guo et al., 2024,  Quantifying the Enhancement of Sarcopenic SkeletalMuscle Preservation Through a Hybrid Exercise Program:Randomized Controlled Trial | The intervention was traditional face-to-face exercise (Tai Chi + strength training). Artificial intelligence (AI) was only used for prediction and analysis of results, not delivered as a core digital health intervention means to patients. |
| Liu et al., 2024,  Graded Progressive Home-Based Resistance Combined with Aerobic Exercise in Community-Dwelling Older Adults with Sarcopenia: A Randomized Controlled Trial | The intervention was traditional “home resistance and aerobic exercise training”,  which does not fall into the category of digital health intervention (DHI) defined by this study. |
| Jyväkorpi et al., 2021,  The sarcopenia and physical frailty in older people: multi-component treatment strategies (SPRINTT) project: description and feasibility of a nutrition intervention in community-dwelling older Europeans | The intervention in this study (SPRINTT trial) was traditional physical activity and nutritional counseling. This does not meet the inclusion criteria for “Digital Health Intervention” (DHI) for this Meta-analysis. |
| Tong et al., 2022,  Study on the effect of health education and whey protein combined with resistance exercise intervention on sarcopenia in middle-aged and elderly people | The intervention was traditional “health education and whey protein combined with resistance exercise”, which does not meet the definition of "Digital Health Intervention" (DHI) for this study. |
| **Reason 4: Other reasons (n=9)** |  |
| Yu et al., 2025,  Sarcopenia prediction model based on machine learning and SHAP values for community-based older adults with cardiovascular disease in China | Lack of data required for the study |
| Chun et al., 2024,  Application study of remote diet and exercise intervention in elderly patients with type 2 diabetes and sarcopenia | Outcome indicators do not match |
| Chang et al., 2025,  The Usability and Effect of a Novel Intelligent Rehabilitation Exergame System on Quality of Life in Frail Older Adults: Prospective Cohort Study | Lack of data required for the study |
| Huang et al., 2024,  Application effect and quality of life impact of evidence-based precision nursing combined with Baduanjin exercise in elderly patients with sarcopenia | Outcome indicators do not match |
| Wang et al., 2023,  Study on the quality of life of elderly patients with sarcopenia using virtual Baduanjin | Lack of data required for the study |
| Liu et al., 2022,  Impact of remote rehabilitation plans on the physical function of elderly patients with sarcopenia | Outcome indicators do not match |
| Zhu et al., 2023,  Intervention study on reducing sedentary behavior on the physical function of elderly patients with sarcopenia | Outcome indicators do not match |
| Yang et al., 2023,  Effect of intervention based on the theory of planned behavior on muscle attenuation and balance ability in elderly people with sarcopenia | Lack of data required for the study |
| Shi et al., 2025,  Social media-based health education plus exercise programme (SHEEP) to improve muscle function among community-dwelling young-old adults with possible sarcopenia: A feasibility study. | Outcome indicators do not match |
